# Exploring the Shift in Structure and Function of Microbial Communities Performing Biological Phosphorus Removal

**DOI:** 10.1371/journal.pone.0161506

**Published:** 2016-08-22

**Authors:** Yanping Mao, Zhiping Wang, Liguan Li, Xiaotao Jiang, Xuxiang Zhang, Hongqiang Ren, Tong Zhang

**Affiliations:** 1 College of Chemistry and Environmental Engineering, Shenzhen University, Shenzhen, China; 2 Environmental Biotechnology Laboratory, Department of Civil Engineering, The University of Hong Kong, Pokfulam Road, Hong Kong, China; 3 School of Environment, Nanjing University, Nanjing, China; Dong-A University, REPUBLIC OF KOREA

## Abstract

A sequencing batch reactor fed mainly by acetate was operated to perform enhanced biological phosphorus removal (EBPR). A short-term pH shock from 7.0 to 6.0 led to a complete loss of phosphate-removing capability and a drastic change of microbial communities. 16S rRNA gene pyrosequencing showed that large proportions of glycogen accumulating organisms (GAOs) (accounted for 16% of bacteria) bloomed, including *Candidatus* Competibacter phosphatis and *Defluviicoccus*-related tetrad-forming organism, causing deteriorated EBPR performance. The EBPR performance recovered with time and the dominant *Candidatus* Accumulibacter (Accumulibacter) clades shifted from Clade IIC to IIA while GAOs populations shrank significantly. The Accumulibacter population variation provided a good opportunity for genome binning using a bi-dimensional coverage method, and a genome of Accumulibacter Clade IIC was well retrieved with over 90% completeness. Comparative genomic analysis demonstrated that Accumulibacter clades had different abilities in nitrogen metabolism and carbon fixation, which shed light on enriching different Accumulibacter populations selectively.

## Introduction

Enhanced biological phosphorus removal (EBPR) is a cost-effective and environmental friendly technology to promote excess phosphorus (P) removal from wastewater. Polyphosphate-accumulating organisms (PAOs) are considered as the contributors for EBPR by accumulating large quantity of phosphorus from the bulk liquid to biosynthesize intracellular polyphosphate. Among the PAOs, a population of microorganisms named *Candidatus* Accumulibacter phosphatis (henceforth referred to Accumulibacter) was readily enriched with acetate as the primary carbon source in sequencing batch reactors (SBRs) [1] and thought to be responsible for EBPR in both lab-scale and full-scale plants [2, 3]. The Accumulibacter lineage can be subdivided into five clades in Type I and seven clades in Type II based on the phylogenetic distance of the gene encoding polyphosphate kinase (*ppk1*) [4–6]. Another population of putative PAOs, members of genus *Tetrasphaera* which can be phylogenetically divided into three distinct clades [7], was detected in many full-scale EBPR plants using glucose or acetate as carbon sources [8]. Besides these two major PAOs, *Gemmatimonas aurantiaca* [9] isolated from an EBPR SBR and *Candidatus* Halomonas phosphatis [10] identified by microautoradiography combined with fluorescence *in situ* hybridization (FISH) in full-scale EBPR plants are also putative PAOs.

Under the alternative anaerobic feast and aerobic famine conditions, PAOs theoretically assimilate anaerobic carbon source by utilizing the aerobically stored polyphosphate. In this process, glycogen-accumulating organisms (GAOs) are considered as competitors to PAOs, as they compete for volatile fatty acids (VFA) under anaerobic condition but do not take up phosphorus in excess of the requirement for growth. GAOs convert VFA to polyhydroxyalkanoates (PHA) under anaerobic condition, then oxidize PHA to CO_2_ or transform to glycogen in the following aerobic phase. This glycogen provides energy and reducing equivalents for the VFA uptake and transformation that will occur in the coming anaerobic period [11, 12]. GAOs are known to be abundant in deteriorated EBPR plants and thought to be responsible for poor phosphorus removal [13]. Two major lineages of GAOs have been intensively studied, *Candidatus* Competibacter phosphatis (henceforth referred to Competibacter) in the γ-*Proteobacteria* class [14, 15] and *Defluvicoccus*-related tetrad-forming organism (TFO) in α-*Proteobacteria* class [16–19], which can be further subdivided into seven subgroups [20] and four clades [13], respectively.

The microbial competition of PAOs and GAOs could be affected by influent C:P ratio [21], carbon substrates [22–24], temperature [25, 26], salinity [27] and pH [28–30]. Among these factors, pH has been considered as the crucial one affecting the energy requirement of VFA uptake by PAOs under anaerobic conditions. PAOs were reported to take up acetate slower than GAOs when pH of the anaerobic zone was less than 7.25 [29, 31]. Moreover, the acidic pH in the aerobic zone will inhibit the growth of PAOs due to the proliferation of GAOs in the EBPR system, resulting in deteriorated EBPR system [28]. It’s been reported that a slight change of pH from 7.0 to 6.5 had led to EBPR deterioration and a drastic change of microbial populations in a SBR [32].

Different phylotypes of PAOs and GAOs were often found to coexist in EBPR systems [6, 20, 33]. Their population dynamics in plants were intensively revealed by FISH [3, 34] and clone libraries [7]. Currently the fast-developed high throughput sequencing has accelerated profiling the EBPR microbial community and functional characteristics [35]. Specific metabolic models have been constructed based on reassembled genomes of EBPR-related bacteria, e.g. *Candidatus* Accumulibacter phosphatis Clade IIA strain UW-1 (referred to CAP IIA UW-1) [36], Clade IA strain UW-2 (referred to CAP IA UW-2) [37], Clade IB strain HKU-1 [38] and Clades IIF, IIC and IC [39], *Tetrasphaera* sp. [8], *Candidatus* Competibacter denitrificans and *Candidatus* Contendobacter odensis [14], and *Defluviicoccus*-related TFOs Clusters I [19] and II [40].

These intensive studies have given a deeper, but still inconclusive, insight into the shift of microbial communities involved in EBPR process under the changes of operational conditions. Different genomic characteristics among uncultured Accumulibacter lineages in EBPR have not been fully uncovered. Therefore this study aims at a) revealing variation of abundances of PAOs and GAOs by using high throughput sequencing, b) uncovering the shift of Accumulibacter clades and c) disclosing the difference of functional potentials among Accumulibacter clades after retrieving another novel genome.

## Materials and Methods

### Sludge sampling and DNA extraction

A cylinder SBR with a working volume of 2 L and 8 cm inner diameter was operated under alternatively anaerobic/aerobic phases at room temperature. The SBR was run at cycles of 6 h (5 min filling, 2.25 h anaerobic phase, 2.75 h aerobic phase, 1 h settling and 5 min withdrawing) with hydraulic retention time of 12 h and solid retention time of about 5 d. The synthetic influent contained (per liter) 15 mg orthophosphate, 14 mg NH_4_^+^-N and 100 mL concentrated carbon solution which consisted of 422 mg sodium acetate, 86 mg glucose and 80 mg yeast extract. The inoculums came from a laboratory reactor performing nitrification stably. The pH was controlled at 7.2 ± 0.1 except at 60^th^ d when accidentally overdosed acidic solution to decrease pH to 6.0 for almost one day (~20 hours).

**Fig 1:**
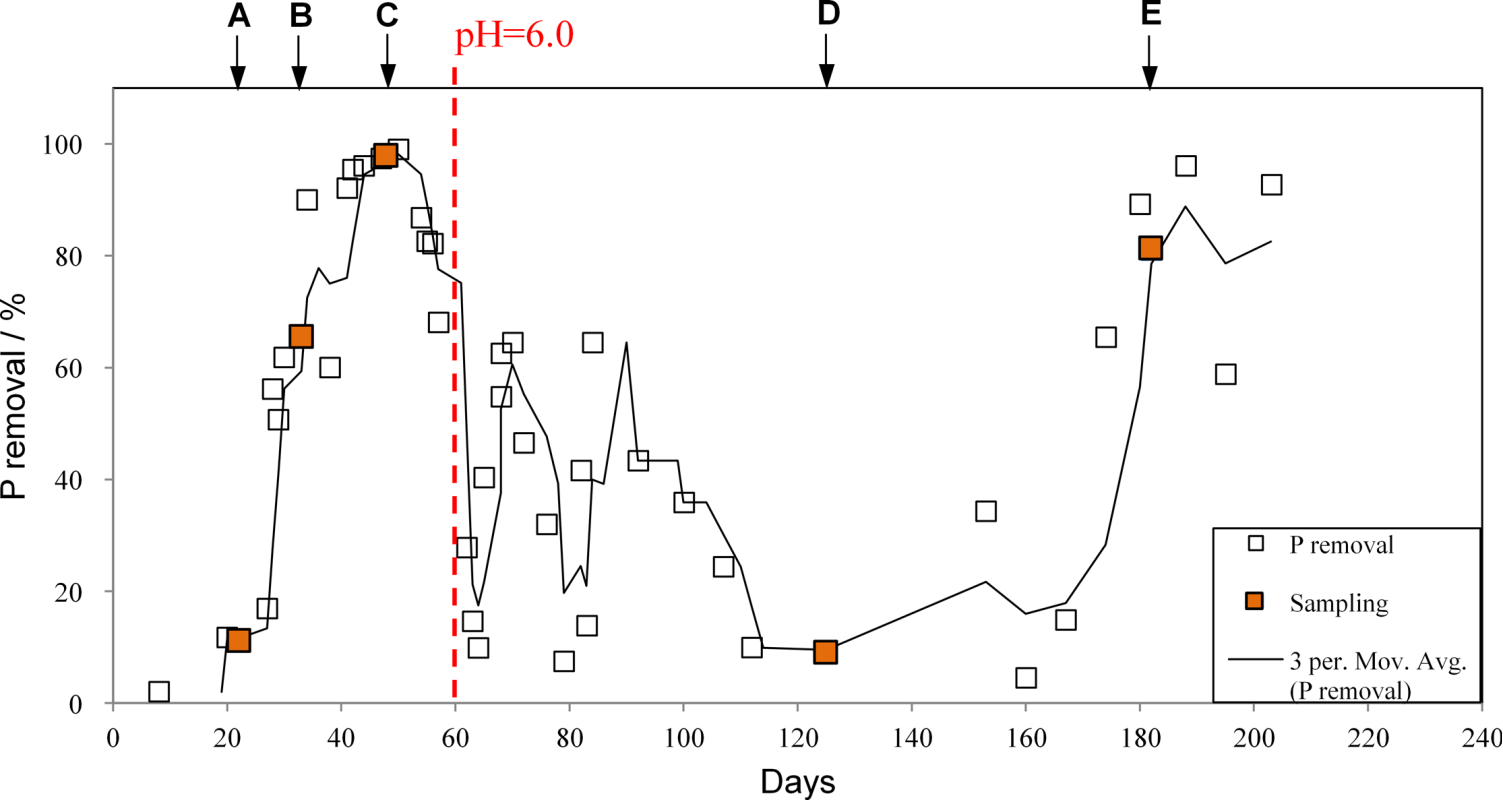
Phosphorus removal and sludge sampling for 16S rRNA gene pyrosequencing from the SBR performing EBPR. The pH of solution in the SBR was maintained at 7.2 ± 0.1 except at 60 d when accidentally overdosed acidic solution to decrease pH to 6.0 for around 20 h.

Sludge samples were taken from the SBR after running for 22 d (A), 33 d (B), 48 d (C), 125 d (D) and 182 d (E) (Fig 1). Total DNA from each sludge sample was extracted in triplicate using Fast DNA Spin kit for Soil (MP Biomedicals, Solon, OH, USA). The concentration and integrity of DNA were measured by NanoDrop^®^ spectrophotometer ND-1000 (Thermo Fisher Scientific, USA) and agarose gel electrophoresis, respectively.

### Bacterial diversity revealed by 16S rRNA gene pyro-tags

16S rRNA genes targeting V3 and V4 regions were amplified from the triplicate DNA extracts of each sample following the procedures specified before [38]. PCR amplicons were purified with a quick-spin Kit (iNtRON, Seoul, Korea), and concentrations were measured by NanoDrop^®^ spectrophotometer ND-1000. The purified amplicons were sent out for pyrosequencing on the Roche 454 FLX Titanium platform at the BGI Company (BGI, Shenzhen, China).

Raw reads were analyzed using Quantitative Insights Into Microbial Ecology (QIIME v. 1.3.0) pipeline [41]. The sequencing data were initially de-multiplexed and separated into different samples based on their nucleotide barcodes. Then, the sequences in each sample were denoised by AmpliconNoise. Chimera checking was performed using Chimera Slayer [42]. The effective reads which we called pyro-tags were produced. Briefly, the pyro-tags were clustered and assigned to operational taxonomic units (OTUs) with 97% similarity cutoff. Taxonomy of representative sequences from each OTU was aligned using the SILVA bacterial database implemented in Mothur platform (v. 1.33.2) [43]. And the diversity indicators including Good’s coverage, ACE and Chao 1 richness estimators were calculated as well.

### Abundances of PAOs and GAOs

Following the methodology published previously [44], an approach was applied to quantify the abundances of PAOs and GAOs using reference 16S rRNA genes (≥ 1200 bp). BLASTN (v.2.2.29+) was used to align pyro-tags to representative PAO 16S rRNA genes from Accumulibacter, *Tetrasphaera* sp., *Candidatus* Halomonas phosphatis and *Gemmatimonas aurantiaca* (S1 Table) and GAO 16S rRNA genes from Competibacter and *Defluvicoccus*-related TFOs (S2 Table) according to the best-hit method. Then, pyro-tags were assigned as PAO-like or GAO-like at 97% similarity and 400 bp alignment length cutoff referring to the above references. To evaluate the reliability of the method, the PAO-like and GAO-like pyro-tags were manually checked online at NCBI to validate the identification.

### Diversity of the Accumulibacter lineage

A published primer set (ACCppk1-254F and ACCppk1-1376R) targeting the Accumulibacter cluster [4] was applied to amplify *ppk*1 gene fragments from A and C DNA samples. The amplified fragments were used for constructing the clone library and then sequencing of positive clones. The obtained DNA sequence was compared to search their closest *ppk1* gene sequences available in GenBank. Then DNA sequences and their closest reference sequences were aligned by MEGA (v.5.02) [45] to construct a phylogenetic tree using neighbor-joining method with a bootstrap of 500. Quantitative real-time PCR (qPCR) assay was adopted to evaluate the relative abundances of Accumulibacter in different clades by using primer sets targeting *ppk1* genes of five specific clades [5].

### Metagenomic sequencing, quality filtering, assembly and gene prediction

The DNA samples from sludges A and C (S1 Fig) were sent to BGI company for paired-end sequencing (2×100 bp) using an Illumina HiSeq 2000 platform. The raw metagenomic sequences were trimmed when having more than 50% nucleotides with the quality scores of lower than 20 [46]. The *de novo* assembled scaffolds (≥ 1 kbp) by CLC Bio Genomics Workbench (v. 6.0.2) [47] were retained for further analysis. Open reading frames (ORFs) were predicted using Prodigal (v. 2.50) [48].

### Genome binning and gene annotation

To bin the genome of dominant Accumulibacter in both A and C microbial communities, a combination of bi-dimensional coverage and tetranucleotide frequency patterns was applied [49]. Scaffold coverage was calculated by mapping the metagenomic reads to the assembled scaffolds using the CLC’s map reads to a reference algorithm with a minimum similarity of 90% over 95% of the read length. The highly conserved 16S rRNA gene which had not been assembled in scaffolds was reconstructed using EMIRGE [50, 51]. The completeness and potential contamination of the draft genome were evaluated according to the well-recognized cluster of orthologous genes (COG)-based [37, 52] and essential single copy genes (ESCG)-based methods [14, 53]. Gene annotation was conducted based on best-hit BLASTP (v.2.2.29+) [54] results again NCBI nr database (downloaded on July 18, 2014).

### Comparative analysis of Accumulibacter genomes

Average nucleotide identity (ANI) between two genomes was calculated using the reciprocal best hits method [55]. Genes of one Accumulibacter genome were compared with the genes of another and vice versa, using BLASTN. The results were screened to remove any alignment that was less than 40% of the gene length. Another in silico genome-to-genome comparison was conducted by using Genome-to-Genome Distance Calculator (GGDC) 2.0 [56] to replace the wet-lab DNA-DNA hybridization (DDH), which in principle was an estimate of the overall similarity between the genomes of two strains.

Following the methods published before [8], the unique and conserved genes in the Accumulibacter genomes were identified by comparing the protein sequences from each Accumulibacter genome against the total proteins in the four available Accumulibacter genomes Clade IIC using BLASTP. Under the criteria of > 50% identical over > 50% of the protein sequence, the genes that cannot hit to any genes from the other Accumulibacter genomes were considered as unique genes, otherwise as non-unique (or conserved) genes.

More detail can be found in S1 Text Supplementary Materials and Methods. The metagenomic sequencing data sets have been deposited in the NCBI Sequence Read Archive (SRA) database under the accession number of SRP041328. The sequences of Accumulibacter Clade IIC HKU-2 draft genome have been deposited in the NCBI Whole Genome Shotgun Database under the accession number of LBIV00000000.

## Results and Discussion

### SBR performance

Five sludge samples performing different P removal efficiencies were extracted for microbial structure comparison. The P removals at the initial stage were below 10% (sludge A), then climbed up to over 60% (sludge B) after 33 d enrichment and achieved almost 100% (sludge C) at 48^th^ d. However, the P removal decreased from the peak due to a short-term pH shock down to pH of 6 at 60^th^ d. Although the pH was recovered to 7.2 ± 0.1 within 1 d, the P removal rates were quite unstable and achieved as low as 9% at 125^th^ d (sludge D). At this moment, the organisms completely lost phosphate-accumulating capability, indicated by the drastic reduction of biomass P content from 7% (C) to 2% (D) and poor P removal in effluent. After two months recovery, the P removal rose again to achieve over 80% at 182^nd^ d (sludge E). Similar pH-effect patterns on EBPR SBR performance have been vigorously studied [32, 57], but the drastic change of microbial population have not been fully uncovered due to certain limitations of molecular technologies and EBPR-related microbial information at that time. In order to reveal the microbial structure variation in the community, DNA samples from A—E were extracted in triplicate for 16S rRNA gene pyrosequencing.

### Microbial community structure

Totally 139,736 pyro-tags targeting 16S rRNA genes in V3 and V4 regions were obtained, with 4,402 to 13,461 sequences in different samples and Good’s coverage of above 98% (S3 Table). The rarefaction curves (S2 Fig) illustrated that the microbial diversities increased from sludge A to E under the EBPR selecting pressure. Pyro-tags showed that the bacteria in sludges A and C mainly belonged to β-*Proteobacteria* and α-*Proteobacteria* classes (S3 Fig), respectively. The representative sequences of OTU 6 and OTU 27 were assigned to Accumulibacter and clustered in Accumulibacter Clade IIC and Clade I 16S rRNA gene homologies, respectively (S4 Fig).

**Fig 2:**
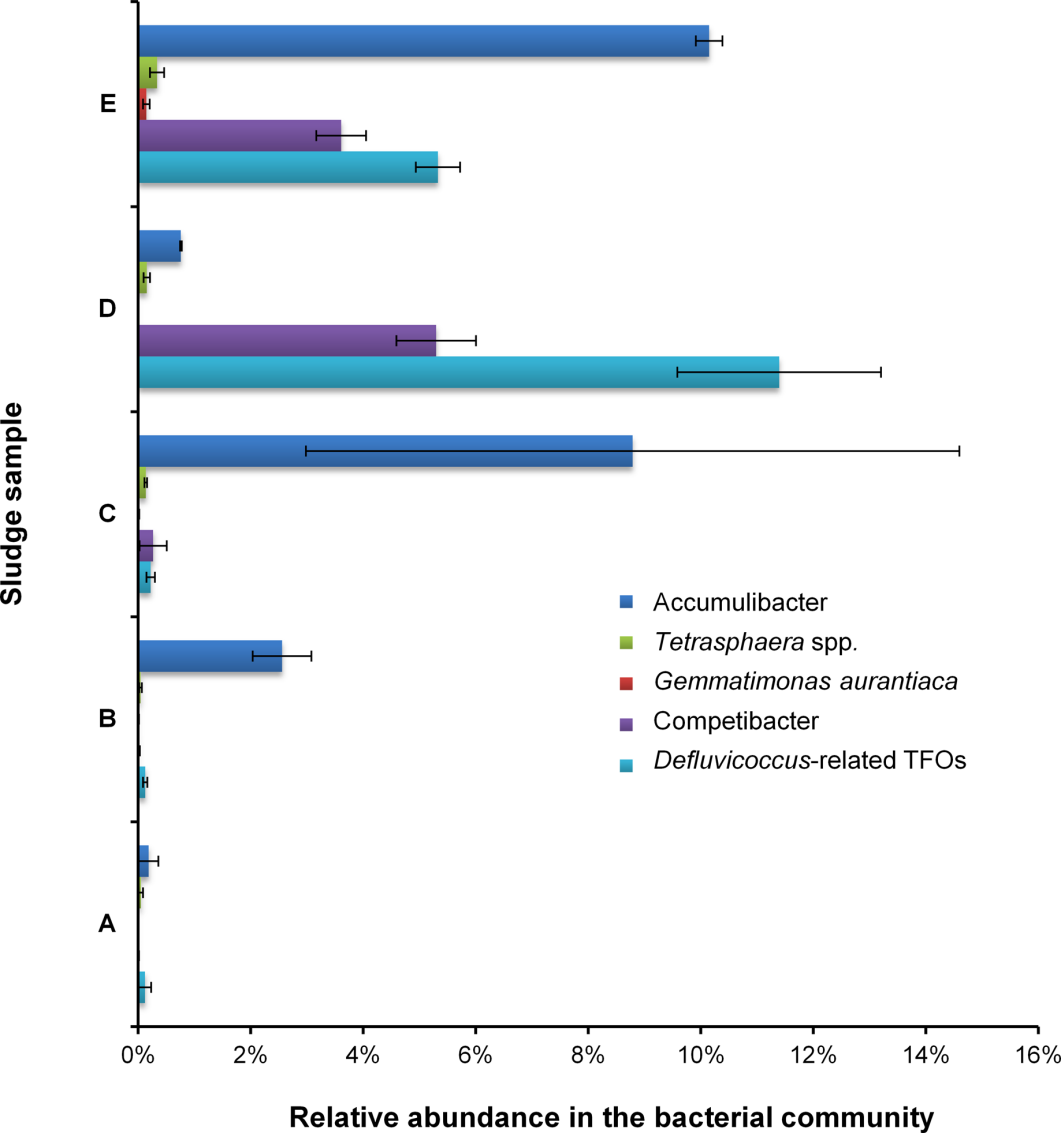
Population dynamics of PAOs and GAOs involved in the SBR. The abundance was calculated based on bacterial pyro-tags which best hit the representative 16S rRNA gene sequences of specific PAO or GAO group with minimum identity of 97% and alignment length cutoff of 400 bp. Standard deviation was calculated from the triplicate pyrosequencing data sets for each sludge sample.

P removal rates were basically proportional to the abundances of PAOs and inversely proportional to the abundances of GAOs. Among the five sludge samples, Accumulibacter dominated the PAO population while there were rare (<0.5%) *Tetrasphaera* sp. (most of which belonged to Clade I), negligible (<0.2%) *Gemmatimonas aurantiaca*, and no *Candidatus* Halomonas phosphatis (Fig 2). Similar to previous studies, the dominance of Accumulibacter was easily identified in EBPR-plants mainly fed by acetate [58]. The small amount of *Tetrasphaera* was probably enriched by utilizing a small proportion of glucose in the influent, since genomic annotation demonstrated that *Tetrasphaera* genomes carried enzymes responsible for glucose assimilation while Accumulibacter genomes did not [8].

In sludges A—C, GAOs were rarely identified. However, large proportions of GAOs including Competibacter and *Defluvicoccus*-related TFOs (dominated by Cluster I) presented in sludges D and E when poor P removal occurred (Fig 2 and S5 Fig). The detected Competibacter in this SBR mainly belonged to subgroup 4 while subgroups 5, 6 and 7 were not identified, consistent with the *in situ* observation from an A/O (anoxic/oxic) SBR fed mainly with acetate [59] and a membrane A/O SBR fed with acetate [20]. The acidic pH shock may led to the proliferation of GAOs and reduction of PAOs, which can be explained by the higher energy demand for PAOs to take up acetate anaerobically and followed by a slower growth rate of PAOs in the aerobic zone [60].

**Fig 3:**
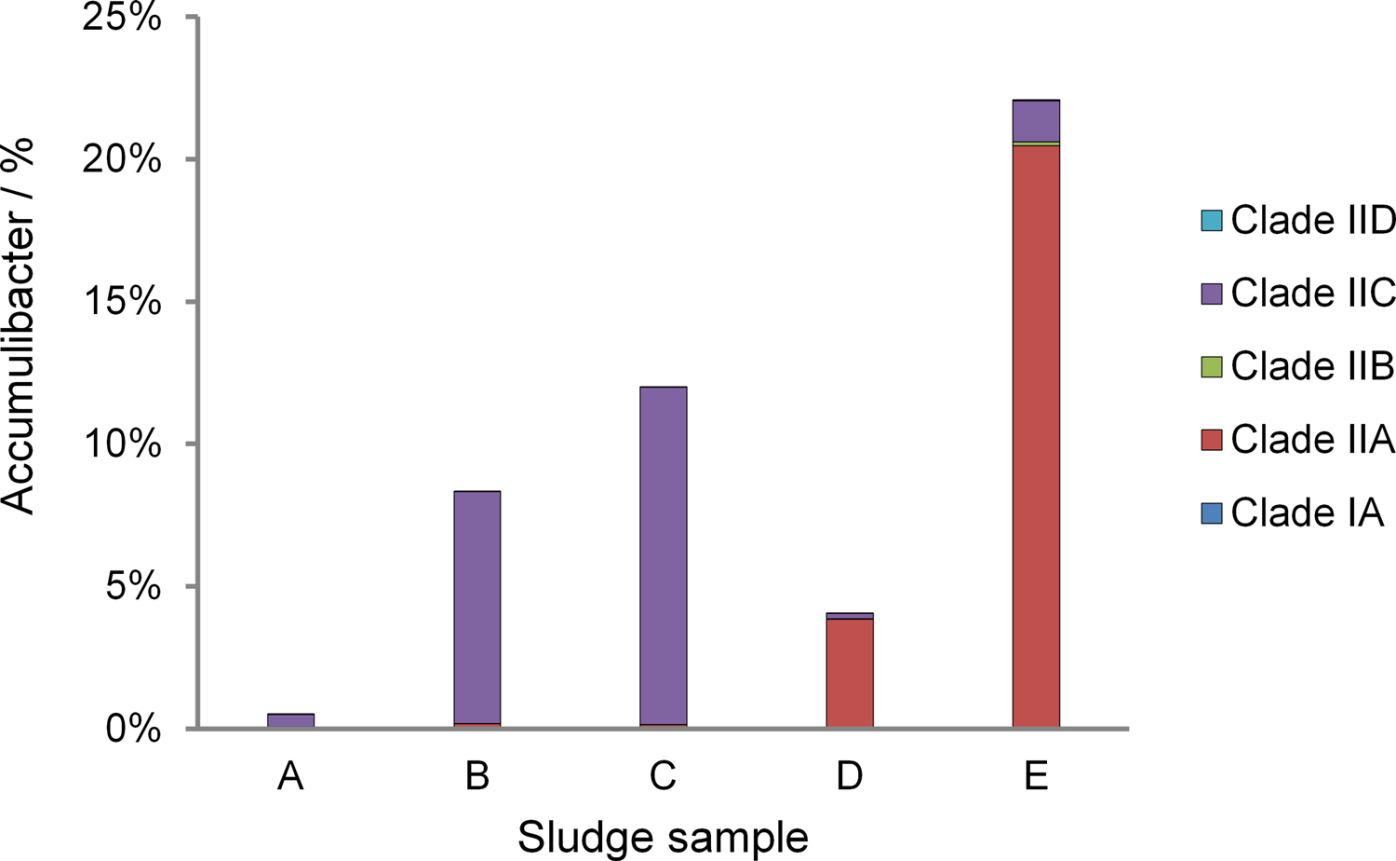
Relative abundances of *ppk1* genes of different clades in the microbial communities. The abundance of Accumulibacter was calculated according to the copy numbers of Accumulibacter and bacterial 16S rRNA genes by qPCR analysis. Meanwhile the 2 copies of *rrn* operon in CAP IIA UW-1 and 4 copies of *rrn* operon in the available bacterial finished genomes have been taken into account. The proportions of different *ppk1* genes in one sample was estimated by the copy numbers obtained from the qPCR assay using primer sets targeting *ppk1* genes of specific clades.

Because 16S rRNA genes cannot reveal the Accumulibacter diversity in a higher resolution, we used the clone library and qPCR analysis of *ppk1* genes to quantify the abundance of specific Accumulibacter clades. The most abundant Accumulibacter belonged to Clade IIC in sludges A, B and C, but shifted to Clade IIA in sludges samples D and E (Fig 3). Therefore, the unstable operation of SBR might reduce the PAO/GAO ratio and induced the shift of Accumulibacter clades. In order to uncover the metabolic characteristics among Accumulibacter clades, another genome in Clade IIC was retrieved from the metagenomic data sets.

### Assembled scaffolds and genome binning

**Fig 4:**
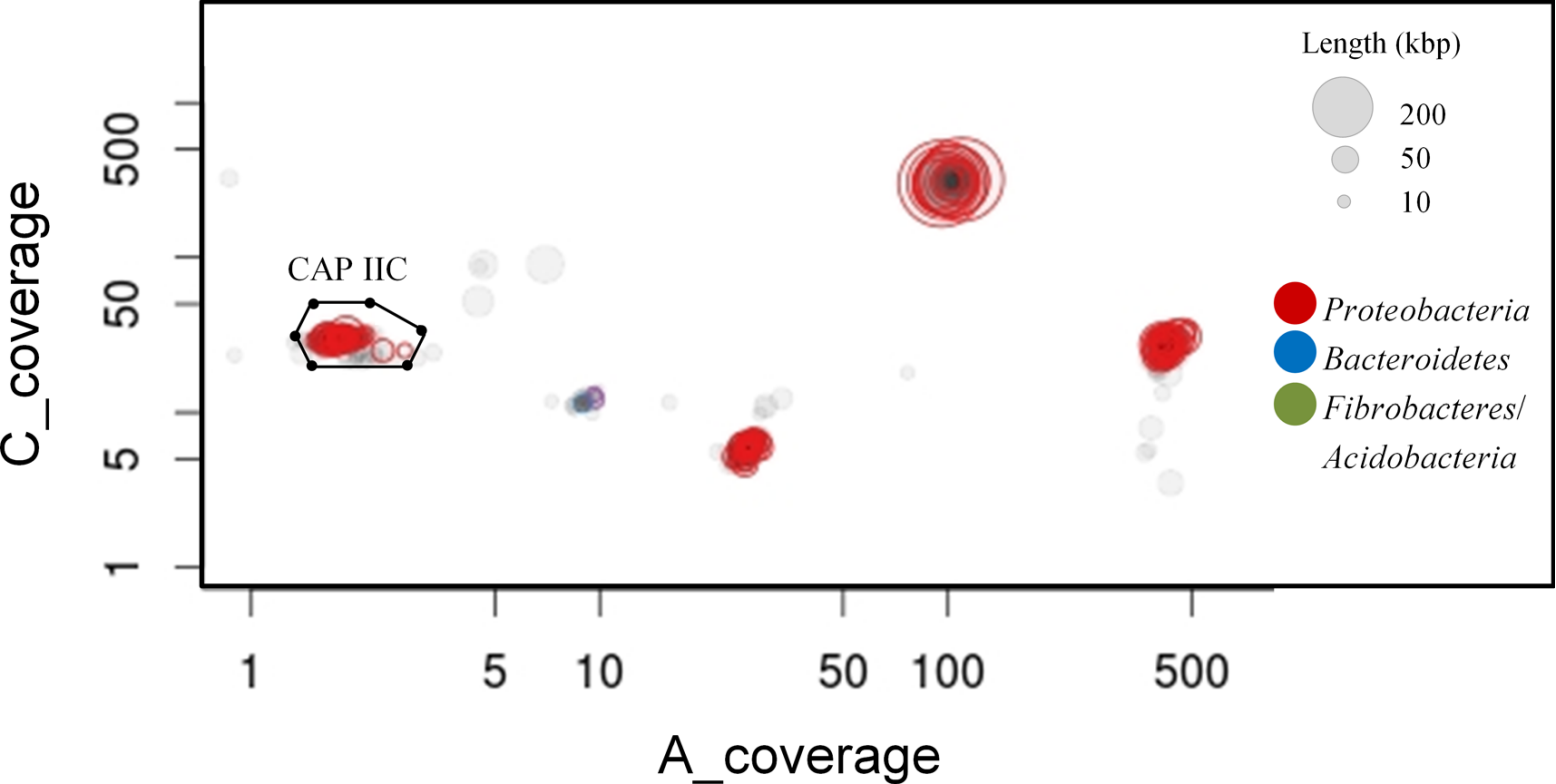
Extraction of the initial genome of Accumulibacter Clade IIC which dominated the PAOs in sludge samples A and C by using the coverage-defined method. Each circle represents an assembled scaffold, with the size proportional to its length and colored by phylum. Only scaffolds ≥ 10 kbp are shown. The box encloses scaffolds representing the initial CAP IIC HKU-2 genome bin.

The two metagenomes from sludge samples A and C resulted in 29.9 and 29.4 million reads after quality filtering respectively which were assembled together using CLC *de novo* algorithm, resulting in 12,801 scaffolds with a minimum length of 1 kbp (Table 1). A genome bin containing 369 scaffolds and 4.3 Mbp in total was retrieved as the Accumulibacter Clade IIC draft genome (defined as CAP IIC HKU-2) mainly according to its coverage in the metagenome (Fig 4), which was consistent with those coverage values estimated from the results of *ppk1* gene clone libraries and qPCR quantification. CAP IIC HKU-2 carried 4,129 genes with an average GC content of 61% (Table 1 and S6 Fig). To determine the completeness of CAP IIC HKU-2, both COG-based and ESCG-based approaches were applied. Among 889 COG functions that were at least present once in all of the seven neighboring reference genomes (S7 Fig), 95 (11%) were missing from CAP IIC draft genome and the completeness was estimated to 89%. However, all 105 ESCG in β-*Proteobacteria* were carried by this draft genome with only 2 ESCGs had two extra copies indicating that CAP IIC HKU-2 should be over 90% completeness with < 2% redundancy.

**Table 1:** Characteristics of assembled scaffolds and the retrieved CAP IIC HKU-2 draft genome.

Characteristics	Assembled scaffolds	CAP IIC HKU-2
Number of scaffolds (≥ 1 kbp)	12,801	369
Total length (bp)	43,617,366	4,262,673
Mean scaffold length (bp)	3,407	11,552
N50	5,466	17,588
Maximum scaffold length (bp)	361,587	65,828
GC content (%)	52	61
ORF number	48,827	4,129
Mean ORF length (bp)	807	922
Total ESCG	\	108
Unique ESCG	\	105
Completeness (COG)	\	89%
Redundancy (COG)	\	6%
*ppk1*	Gene_2225 (Scaffold_5930)	Gene_2225 (Scaffold_5930)
16S rRNA gene ^a^	\	1,567 bp

^a^ 16S rRNA gene was reconstructed by EMIRGE method [50].

**Fig 5:**
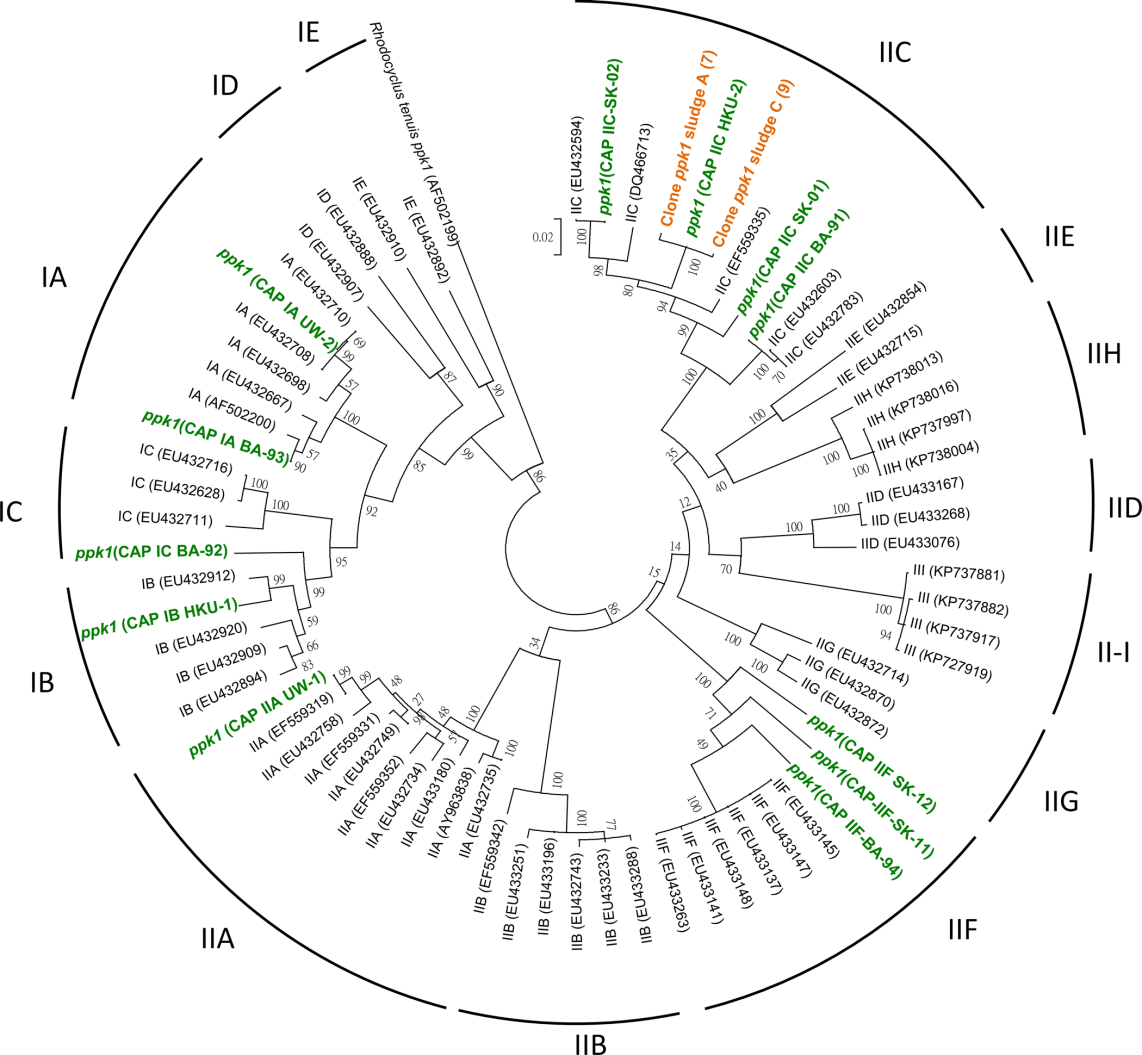
Maximum likelihood phylogenetic tree of Accumulibacter *ppk1* gene sequences. The *ppk1* genes of the reconstructed Accumulibacter genomes are indicated in green, while those from clone library of sludges A and C are colored in orange. Seven and nine partial *ppk1* gene sequences with 99% identity were obtained from sludge A and C respectively. Reference sequences attached with their accession numbers are extracted from NCBI database. Node labels refer to bootstrap support values and *Rhodocyclus tenuis ppk1* gene is employed as the outgroup sequence.

A *ppk1* homolog was identified in a long scaffold (scaffold_5930, 22,567 bp) of CAP IIC HKU-2 draft genome sharing 99% nucleotide identity with a Clade IIC *ppk1* identified previously from *Candidatus* Accumulibacter sp. SK-02 (GenBank EX188565) [39], and clustered with other Clade IIC *ppk1* gene sequences (Fig 5). Moreover, an almost full length of 16S rRNA gene (1,567 bp) clustering in Clade IIC&IID was reconstructed by using EMIRGE approach (S4 Fig). As 16S rRNA gene and *ppk1* gene were originally used to define the Accumulibacter lineage and Accumulibacter clade phylogeny, the reconstruction of the 16S rRNA gene and the presence of a Clade IIC *ppk1* homolog within the genome bin provided additional confidence on taxonomy of Clade IIC HKU-2.

### Comparative analyses of Accumulibacter genomes

**Fig 6:**
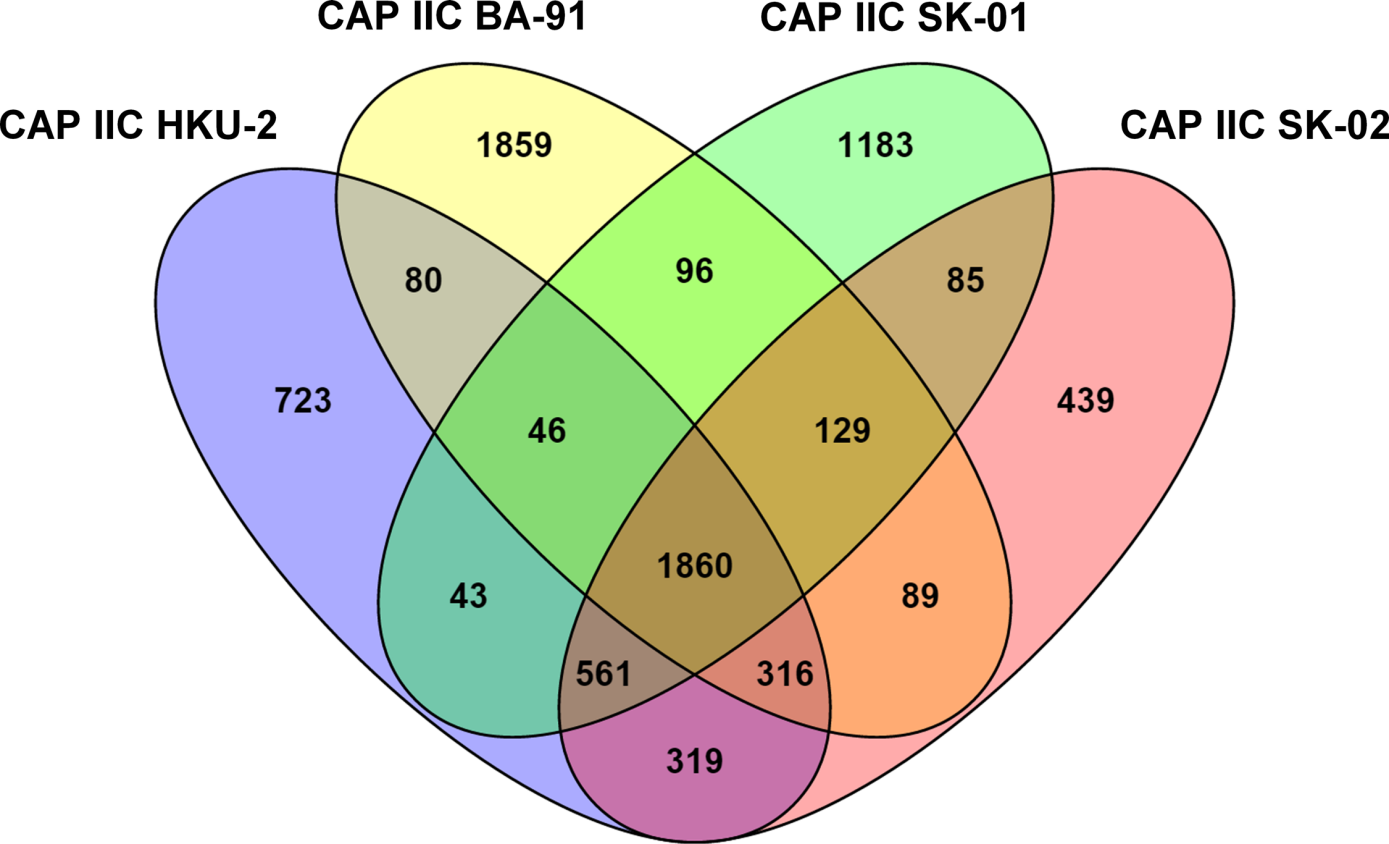
Venn diagram of conserved and unique genes for four Accumulibacter genomes of Clade IIC.

To determine the similarity of Accumulibacter genomes, ANI between two genomes were calculated by the well-recognized reciprocal best hit analysis. The results showed that ANI between two Accumulibacter genomes from different types, such as IIA UW-1 and IA UW-2, were below 81%, while the ANI between two genomes from the same type, such as IIA UW-1 and IIC HKU-2, were ranged in 79%–99% (Table 2). The Accumulibacter Clade IIC genomes, HKU-2 and SK-02 had an ANI score of 99%, which indicated that they were likely strains of the same species [55]. However, both genomes contained large sets of different genes (892 and 742 genes respectively) (Fig 6), which was similar to CAP IA UW-2 and CAP IA BA-93 with a high ANI score of 99% but large unique genes greater than that reported for strains in some genera. The in silico genome-to-genome distance calculation illustrated that the probability for them from the same sub-species (DDH > 79%) is 63%, while those for CAP IA UW-2 and CAP IA BA-93 was 43%. Therefore, the true similarity between the two genomes requires further analysis of shared gene complement [39].

**Table 2:** The ANI and orthologous genes shared by each pair of Accumulibacter genomes.

Genome	IA UW-2	IA BA-93	IB HKU-1	IC BA-92	IIA UW-1	IIC BA-91	IIC SK-01	IIC SK-02	IIC HKU-2	IIF BA-94	IIF SK-11	IIF SK-12
IA UW-2	\	99%	86%	86%	81%	80%	80%	80%	80%	80%	80%	79%
IA BA-93	4801	\	86%	86%	81%	80%	80%	80%	80%	80%	80%	79%
IB HKU-1	2395	2756	\	91%	81%	80%	80%	80%	80%	80%	79%	79%
IC BA-92	3296	3766	3029	\	81%	79%	80%	79%	79%	80%	79%	79%
IIA UW-1	1685	1925	1249	1649	\	81%	82%	81%	81%	81%	82%	81%
IIC BA-91	772	869	597	758	1243	\	89%	88%	88%	80%	79%	79%
IIC SK-01	1184	1326	889	1133	1757	2024	\	94%	94%	80%	80%	79%
IIC SK-02	1155	1327	937	1169	1853	2497	3738	\	99%	80%	79%	79%
IIC HKU-2	1113	1274	2395	1078	1733	2237	3464	4598	\	80%	80%	79%
IIF BA-94	916	1017	670	787	1166	491	809	782	701	\	88%	88%
IIF SK-11	946	1052	680	841	1356	549	907	888	813	1313	\	86%
IIF SK-12	1239	1424	986	1153	1818	752	1151	1208	1114	1770	2279	\

The conserved genes from all four Accumulibacter genomes of Clade IIC constitute 1,860 sequences (Fig 6) including those encoding enzymes responsible for the central metabolic pathways of glycolysis through Embden-Meyerhof-Parnas (EMP) pathway, the tricarboxylic acid (TCA) cycle, acetate uptake, PHA synthesis, polyphosphate metabolism, transportation of orthophosphate and assimilatory sulfate reduction. The unique genes constitute from 439 to 1,859 sequences. Different from Accumulibacter genomes of other clades, four of the Accumulibacter Clade IIC genomes carry the respiratory nitrate reductase gene subunits (*narGHIJ*) that initiated a prokaryotic denitrification pathway [61].

The principal coordinates analysis (PCoA) results based on COG categories (S7 Fig) demonstrated the Accumulibacter genomes were closer to each other than to their neighboring genomes. Each gene inventory of the 12 Accumulibacter genomes were mapped to functional categories in COG database respectively, which comprises those encoding enzymes for central metabolic pathways. According to the COG distribution profile (S8 Fig), majority pertained to COGs of three essential functional categories, including ‘signal transduction mechanisms’ (13.0%), ‘general function prediction only’ (10.4%), as well as ‘amino acid transport and metabolism’ (7.8%).

Additionally, comparing the genomic information of CAP IIC HKU-2 and CAP IIA UW-1, we found that CAP IIA UW-1 had genes required for carbon fixation, however, the key genes required for the Calvin cycle, ribulose-1,5-bisphosphate carboxylase/oxygenase (*rubisco*) and ribulose-phosphate 3-epimerase (*Rpe*) appeared to be absent from CAP IIC HKU-2. This implied that Accumulibacter Clade IIA might survive in organic carbon deficient conditions when occurring proliferation of GAOs which assimilated VFA to outcompete PAOs. The genomic difference may stimulate the shift of Accumulibacter clades in the SBR but it needs further experimental validations.

## Conclusions

This study revealed the population dynamics of PAOs and GAOs involved in the EBPR process. The proliferation of GAOs may induce the shift of dominant Accumulibacter clades from Clade IIC to IIA. In order to find the clues for explaining the shift, another Accumulibacter genome in Clade IIC was retrieved with over 90% completeness. Comparative analyses of EBPR metabolic pathways uncovered that the key genes for carbon fixation appeared in CAP IIA UW-1 were absent in CAP IIC HKU-2, which may help explain the shift of Accumulibacter clades and provide new insights for selective enrichment of the significant PAOs.

## Supporting Information

S1 Fig Phosphorus removal and TOC removal of the sequencing batch reactor. Sludge samples from A and C were collected for metagenomic sequencing.
(PDF)

S2 Fig Rarefaction curves of sludge samples for 16S rRNA gene pyrosequencing with 97% similarity. (PDF)

S3 Fig Relative abundances of bacterial populations in the dominant classes at different stages of the SBR. (PDF)

S4 Fig Phylogenetic tree of 16S rRNA genes from Accumulibacter draft genomes, representative pyro-tags of the OTU groups assigned to Accumulibacters and reference sequences downloaded from GenBank. *Rhodocyclus tenuis* was recruited as the out-group species.
(PDF)

S5 Fig Principal components analysis of five sludge samples extracted from the SBR according to bacterial abundance involved in EBPR process. (PDF)

S6 Fig Binning of CAP IIC HKU-2 draft genome by using differential coverage of metagenomics data sets from sludges A and C. (PDF)

S7 Fig Principal coordinates analysis of Accumulibacter genomes (green dots) and its six neighboring finished genomes (orange dots) in family of *Rhodocyclaceae* using the Euclidean similarity metric based upon their annotated COG categories. (PDF)

S8 Fig COG profile of the 12 Accumulibacter genomes. (PDF)

S1 Table Reference 16S rRNA gene sequences of representative PAOs. (PDF)

S2 Table Reference 16S rRNA gene sequences of representative GAOs. (PDF)

S3 Table Effective 16S rRNA gene pyrosequencing reads generation and microbial diversity metrics. (PDF)

S1 Text Supplementary Materials and Methods. (PDF)

## References

[1] Hesselmann RPX, Werlen C, Hahn D, van der Meer JR, Zehnder AJB. Enrichment, phylogenetic analysis and detection of a bacterium that performs enhanced biological phosphate removal in activated sludge. Syst Appl Microbiol. 1999;22(3):454–65. 10.1016/s0723-2020(99)80055-1 10553298

[2] Oehmen A, Lemos P, Carvalho G, Yuan Z, Keller J, Blackall L, et al Advances in enhanced biological phosphorus removal: from micro to macro scale. Water Res. 2007;41(11):2271–300. 10.1016/j.watres.2007.02.030 17434562

[3] Mielczarek AT, Nguyen HT, Nielsen JL, Nielsen PH. Population dynamics of bacteria involved in enhanced biological phosphorus removal in Danish wastewater treatment plants. Water Res. 2013;47(4):1529–44. 10.1016/j.watres.2012.12.003 23317522

[4] McMahon KD, Yilmaz S, He S, Gall DL, Jenkins D, Keasling JD. Polyphosphate kinase genes from full-scale activated sludge plants. Appl Microbiol Biotechnol. 2007;77(1):167–73. 10.1007/s00253-007-1122-6 17671784

[5] He S, Gall DL, McMahon KD. "*Candidatus* Accumulibacter" population structure in enhanced biological phosphorus removal sludges as revealed by polyphosphate kinase genes. Appl Environ Microbiol. 2007;73(18):5865–74. 10.1128/aem.01207-07 17675445 PMC2074919

[6] Peterson SB, Warnecke F, Madejska J, McMahon KD, Hugenholtz P. Environmental distribution and population biology of *Candidatus* Accumulibacter, a primary agent of biological phosphorus removal. Environ. Microbiol. 2008;10(10):2692–703. 10.1111/j.1462-2920.2008.01690.x 18643843 PMC2561248

[7] Nguyen HTT, Le VQ, Hansen AA, Nielsen JL, Nielsen PH. High diversity and abundance of putative polyphosphate-accumulating *Tetrasphaera*-related bacteria in activated sludge systems. FEMS Microbiol Ecol. 2011;76(2):256–67. 10.1111/j.1574-6941.2011.01049.x 21231938

[8] Kristiansen R, Nguyen HT, Saunders AM, Nielsen JL, Wimmer R, Le VQ, et al A metabolic model for members of the genus *Tetrasphaera* involved in enhanced biological phosphorus removal. ISME J. 2013;7(3):543–54. 10.1038/ismej.2012.136 23178666 PMC3578573

[9] Zhang H. *Gemmatimonas aurantiaca* gen. nov., sp. nov., a Gram-negative, aerobic, polyphosphate-accumulating micro-organism, the first cultured representative of the new bacterial phylum *Gemmatimonadetes* phyl. nov. Int J Syst Evol Microbiol. 2003;53(4):1155–63. 10.1099/ijs.0.02520-0 12892144

[10] Nguyen HT, Nielsen JL, Nielsen PH. '*Candidatus* Halomonas phosphatis', a novel polyphosphate-accumulating organism in full-scale enhanced biological phosphorus removal plants. Environ Microbiol. 2012;14(10):2826–37. 10.1111/j.1462-2920.2012.02826.x 22827168

[11] Filipe CDM, Daigger GT Jr. CPLG. A metabolic model for acetate uptake under anaerobic conditions by glycogen accumulating organisms: stoichiometry, kinetics, and the effect of pH. Biotechnol Bioeng. 2001;76(1):17–31. 10.1002/bit.1022 11400103

[12] Zeng RJ, van Loosdrecht MCM, Yuan Z, Keller Jr. Metabolic model for glycogen-accumulating organisms in anaerobic/aerobic activated sludge systems. Biotechnol Bioeng. 2003;81(1):92–105. 10.1002/bit.10455 12432585

[13] Meyer RL. Putative glycogen-accumulating organisms belonging to the *Alphaproteobacteria* identified through rRNA-based stable isotope probing. Microbiology. 2006;152(2):419–29. 10.1099/mic.0.28445-0 16436430

[14] McIlroy SJ, Albertsen M, Andresen EK, Saunders AM, Kristiansen R, Stokholm-Bjerregaard M, et al '*Candidatus* Competibacter'-lineage genomes retrieved from metagenomes reveal functional metabolic diversity. ISME J. 2014;8:613–24. 10.1038/ismej.2013.162 24173461 PMC3930307

[15] Crocetti GR, Banfield JF, Keller J, Bond PL, Blackall LL. Glycogen-accumulating organisms in laboratory-scale and full-scale wastewater treatment processes. Microbiology. 2002;148:3353–64. 10.1099/00221287-148-11-3353 12427927

[16] Mcllroy S, Seviour RJ. Elucidating further phylogenetic diversity among the *Defluviicoccus*-related glycogen-accumulating organisms in activated sludge. Environ Microbiol Rep. 2009;1(6):563–8. 10.1111/j.1758-2229.2009.00082.x 23765935

[17] Wong MT, Tan FM, Ng WJ, Liu WT. Identification and occurrence of tetrad-forming *Alphaproteobacteria* in anaerobic-aerobic activated sludge processes. Microbiology. 2004;150(11):3741–8. 10.1099/mic.0.27291-0 15528660

[18] Nielsen AT, Liu W-T, Filipe C Jr. LG, Molin S, Stahl DA. Identification of a novel group of bacteria in sludge from a deteriorated biological phosphorus removal reactor. Appl Environ Microbiol. 1999;65(3):1251–8. 10049891 10.1128/aem.65.3.1251-1258.1999 PMC91172

[19] Nobu MK, Tamaki H, Kubota K, Liu WT. Metagenomic characterization of '*Candidatus* Defluviicoccus tetraformis strain TFO71,' a tetrad-forming organism, predominant in an anaerobic-aerobic membrane bioreactor with deteriorated biological phosphorus removal. Environ Microbiol. 2014;16(9):2739–51. 10.1111/1462-2920.12383 24428681

[20] Kong Y, Ong SL, Ng WJ, Liu W-T. Diversity and distribution of a deeply branched novel proteobacterial group found in anaerobic-aerobic activated sludge processes. Environ Microbiol. 2002;4(11):753–7. 12460283 10.1046/j.1462-2920.2002.00357.x

[21] Ong YH, Chua ASM, Lee BP, Ngoh GC. Long-term performance evaluation of EBPR process in tropical climate: start-up, process stability, and the effect of operational pH and influent C:P ratio. Water Sci Technol. 2013;67(2):340–6. 10.2166/wst.2012.552 23168633

[22] Chen Y, Randall AA, McCue T. The efficiency of enhanced biological phosphorus removal from real wastewater affected by different ratios of acetic to propionic acid. Water Res. 2004;38:27–36. 10.1016/j.watres.2003.08.025 14630100

[23] Chen Y, Liu Y, Zhou Q, Gu G. Enhanced phosphorus biological removal from wastewater-effect of microorganism acclimatization with different ratios of short-chain fatty acids mixture. Biochem Eng J. 2006;27(1):24–32.

[24] Wang Y, Jiang F, Zhang Z, Xing M, Lu Z, Wu M, et al The long-term effect of carbon source on the competition between polyphosphorus accumulating organisms and glycogen accumulating organism in a continuous plug-flow anaerobic/aerobic (A/O) process. Biores Technol. 2010;101:98–104. 10.1016/j.biortech.2009.07.085 19729302

[25] Lopez-Vazquez CM, Song Y-I, Hooijmans CM, Brdjanovic D, Moussa MS, Gijzen HJ, et al Short-term temperature effects on the anaerobic metabolism of glycogen accumulating organisms. Biotechnol Bioeng. 2007;97(3):483–95. 10.1002/bit.21302 17171717

[26] Pansward T, Doungchai A, Anotai J. Temperature effect on microbial community of enhanced biological phosphorus removal system. Water Res. 2003;37:409–15. 12502069 10.1016/s0043-1354(02)00286-5

[27] Uygur A, Kargı F. Salt inhibition on biological nutrient removal from saline wastewater in a sequencing batch reactor. Enzyme Microb Technol. 2004;34:313–8. 10.1016/j.enzmictec.2003.11.010

[28] Filipe CDM, Daigger GT Jr. CPLG. Effects of pH on the rates of aerobic metabolism of phosphate-accumulating and glycogen-accumulating organisms. Water Environ Res. 2001;73(2):213–22. 11563381 10.2175/106143001x139191

[29] Filipe CDM, Daigger GT Jr. CPLG. pH as a key factor in the competition between glycogen-accumulating organisms and phosphorus-accumulating organisms. Water Environ Res. 2001;73(2):223–32. 11563382 10.2175/106143001x139209

[30] Lopez-Vazquez CM, Oehmen A, Hooijmans CM, Brdjanovic D, Gijzen HJ, Yuan Z, et al Modeling the PAO–GAO competition: Effects of carbon source, pH and temperature. Water Res. 2009;43(2):450–62. 10.1016/j.watres.2008.10.032 19022471

[31] Filipe CDM, Daigger GT Jr. CPLG. A metabolic model for acetate uptake under anaerobic conditions by glycogen accumulating organisms: stoichiometry, kinetics, and the effects of pH. Biotechnol Bioeng. 2001;76(1):17–31. 11400103 10.1002/bit.1022

[32] Zhang T, Liu Y, Fang HHP. Effect of pH change on the performance and microbial community of enhanced biological phosphate removal process. Biotechnol Bioeng. 2005;92(2):173–82. 10.1002/bit.20589 15962340

[33] Kong Y, Xia Y, Nielsen JL, Nielsen PH. Ecophysiology of a group of uncultured Gammaproteobacterial glycogen-accumulating organisms in full-scale enhanced biological phosphorus removal wastewater treatment plants. Environ Microbiol. 2006;8(3):479–89. 10.1111/j.1462-2920.2005.00914.x 16478454

[34] Carvalho G, Lemos PC, Oehmen A, Reis MA. Denitrifying phosphorus removal: linking the process performance with the microbial community structure. Water Res. 2007;41(19):4383–96. 10.1016/j.watres.2007.06.065 17669460

[35] Albertsen M, Hansen LBS, Saunders AM, Nielsen PH, Nielsen KL. A metagenome of a full-scale microbial community carrying out enhanced biological phosphorus removal. ISME J. 2012;6(6):1094–106. 10.1038/ismej.2011.176 22170425 PMC3358022

[36] Martín HG, Ivanova N, Kunin V, Warnecke F, Barry KW, McHardy AC, et al Metagenomic analysis of two enhanced biological phosphorus removal (EBPR) sludge communities. Nat Biotechnol. 2006;24(10):1263–9. 10.1038/nbt1247 16998472

[37] Flowers JJ, He S, Malfatti S, del Rio TG, Tringe SG, Hugenholtz P, et al Comparative genomics of two '*Candidatus* Accumulibacter' clades performing biological phosphorus removal. ISME J. 2013;7(12):2301–14. 10.1038/ismej.2013.117 23887171 PMC3834850

[38] Mao Y, Yu K, Xia Y, Chao Y, Zhang T. Genome reconstruction and gene expression of “*Candidatus* Accumulibacter phosphatis” Clade IB performing biological phosphorus removal. Environ Sci Technol. 2014;48(17):10363–71. 10.1021/es502642b 25089581

[39] Skennerton CT, Barr JJ, Slater FR, Bond PL, Tyson GW. Expanding our view of genomic diversity in *Candidatus* Accumulibacter clades. Environ Microbiol. 2015;17(5):1574–85. 10.1111/1462-2920.12582 25088527

[40] Wang Z, Guo F, Mao Y, Xia Y, Zhang T. Metabolic characteristics of a glycogen-accumulating organism in *Defluviicoccus* cluster II revealed by comparative genomics. Microb Ecol. 2014;68(4):716–28. 10.1007/s00248-014-0440-3 24889288

[41] Caporaso JG, Kuczynski J, Stombaugh J, Bittinger K, Bushman FD, Costello EK, et al QIIME allows analysis of high-throughput community sequencing data. Nat Methods. 2010;7(5):335–6. 10.1038/nmeth.f.303 20383131 PMC3156573

[42] Haas BJ, Gevers D, Earl AM, Feldgarden M, Ward DV, Giannoukos G, et al Chimeric 16S rRNA sequence formation and detection in Sanger and 454-pyrosequenced PCR amplicons. Genome Res. 2011;21(3):494–504. 10.1101/gr.112730.110 21212162 PMC3044863

[43] Schloss PD, Westcott SL, Ryabin T, Hall JR, Hartmann M, Hollister EB, et al Introducing mothur: open-source, platform-independent, community-supported software for describing and comparing microbial communities. Appl Environ Microbiol. 2009;75(23):7537–41. 10.1128/AEM.01541-09 19801464 PMC2786419

[44] Leininger S, Urich T, Schloter M, Schwark L, Qi J, Nicol GW, et al Archaea predominate among ammonia-oxidizing prokaryotes in soils. Nature. 2006;442(7104):806–9. 10.1038/nature04983 16915287

[45] Tamura K, Peterson D, Peterson N, Stecher G, Nei M, Kumar S. MEGA5: molecular evolutionary genetics analysis using maximum likelihood, evolutionary distance, and maximum parsimony methods. Mol Bio Evol. 2011;28(10):2731–9. 10.1093/molbev/msr121 21546353 PMC3203626

[46] Qin J, Li R, Raes J, Arumugam M, Burgdorf KS, Manichanh C, et al A human gut microbial gene catalogue established by metagenomic sequencing. Nature. 2010;464(7285):59–65. 10.1038/nature08821 20203603 PMC3779803

[47] Zerbino DR, Birney E. Velvet: algorithms for de novo short read assembly using de Bruijn graphs. Genome Res. 2008;18(5):821–9. 10.1101/gr.074492.107 18349386 PMC2336801

[48] Hyatt D, Chen G-L, LoCascio PF, Land ML, Larimer FW, Hauser LJ. Prodigal: prokaryotic gene recognition and translation initiation site identification. BMC bioinformatics. 2010;11(1):119 10.1186/1471-2105-11-119 20211023 PMC2848648

[49] Albertsen M, Hugenholtz P, Skarshewski A, Nielsen KL, Tyson GW, Nielsen PH. Genome sequences of rare, uncultured bacteria obtained by differential coverage binning of multiple metagenomes. Nat Biotechnol. 2013;31(6):533–8. 10.1038/nbt.2579 23707974

[50] Miller CS, Baker BJ, Thomas BC, Singer SW, Banfield JF. EMIRGE: reconstruction of full-length ribosomal genes from microbial community short read sequencing data. Genome Bio. 2011;12(5):R44 10.1186/gb-2011-12-5-r44 21595876 PMC3219967

[51] Miller CS, Handley KM, Wrighton KC, Frischkorn KR, Thomas BC, Banfield JF. Short-read assembly of full-length 16S amplicons reveals bacterial diversity in subsurface sediments. PLoS One. 2013;8(2):e56018 10.1371/journal.pone.0056018 23405248 PMC3566076

[52] Hess M, Sczyrba A, Egan R, Kim TW, Chokhawala H, Schroth G, et al Metagenomic discovery of biomass-degrading genes and genomes from cow rumen-supporting information. Science. 2011;331(6016):463–7. 10.1126/science.1200387 21273488

[53] Dupont CL, Rusch DB, Yooseph S, Lombardo MJ, Richter RA, Valas R, et al Genomic insights to SAR86, an abundant and uncultivated marine bacterial lineage. ISME J. 2012;6(6):1186–99. 10.1038/ismej.2011.189 22170421 PMC3358033

[54] Altschul SF, Madden TL, Schäffer AA, Zhang J, Zhang Z, Miller W, et al Gapped BLAST and PSI-BLAST: a new generation of protein database search programs. Nuc Aci Res. 1997;25(17):3389–402. 10.1093/nar/25.17.3389 PMC146917 9254694

[55] Richter M, Rosselló-Móra R. Shifting the genomic gold standard for the prokaryotic species definition. Proc Natl Acad Sci U S A. 2009;106(45):19126–31. 10.1073/pnas.0906412106 19855009 PMC2776425

[56] Meier-Kolthoff JP, Auch AF, Klenk H-P, Göker M. Genome sequence-based species delimitation with confidence intervals and improved distance functions. BMC Bioinformatics. 2013;14(60):14 10.1186/1471-2105-14-60 23432962 PMC3665452

[57] Oehmen A, Teresa Vives M, Lu H, Yuan Z, Keller J. The effect of pH on the competition between polyphosphate-accumulating organisms and glycogen-accumulating organisms. Water Res. 2005;39(15):3727–37. 10.1016/j.watres.2005.06.031 16098556

[58] He S, Gu AZ, McMahon KD. Fine-scale differences between Accumulibacter-like bacteria in enhanced biological phosphorus removal activated sludge. Water Sci Tech. 2006;54(1):111–7. 10.2166/wst.2006.378 16898143

[59] Liu W-T, Nielsen AT, Wu J-H, Tsai C-S, Matsuo Y, Molin S. *In situ* identification of polyphosphate- and polyhydroxyalkanoate-accumulating traits for microbial populations in a biological phosphorus process. Environ Microbiol. 2001;3(2):110–22. 10.1046/j.1462-2920.2001.00164.x 11321541

[60] Liu W-T, Mino T, Matsuo T, Nakamura K. Biological phosphorus removal processes—Effect of pH on anaerobic substrate metabolism. Water Sci Technol. 1996;34(1–2):25–32. 10.1016/0273-1223(96)00491-X

[61] Tavares P, Pereira A, Moura J, Moura I. Metalloenzymes of the denitrification pathway. J Inorg Biochem. 2007;100(12):2087–100. 10.1016/j.jinorgbio.2006.09.003 17070915

