# Supplementary material for: Exploring the Shift in Structure and Function of Microbial Communities Performing Biological Phosphorus Removal
Source: PLoS One. 2016 Aug 22;11(8):e0161506. doi: 10.1371/journal.pone.0161506 (PMC4993488; doi:10.1371/journal.pone.0161506)
Supplement: S1 Fig — Sludge samples from A and C were collected for metagenomic sequencing. (PDF) [file pone.0161506.s001.pdf]

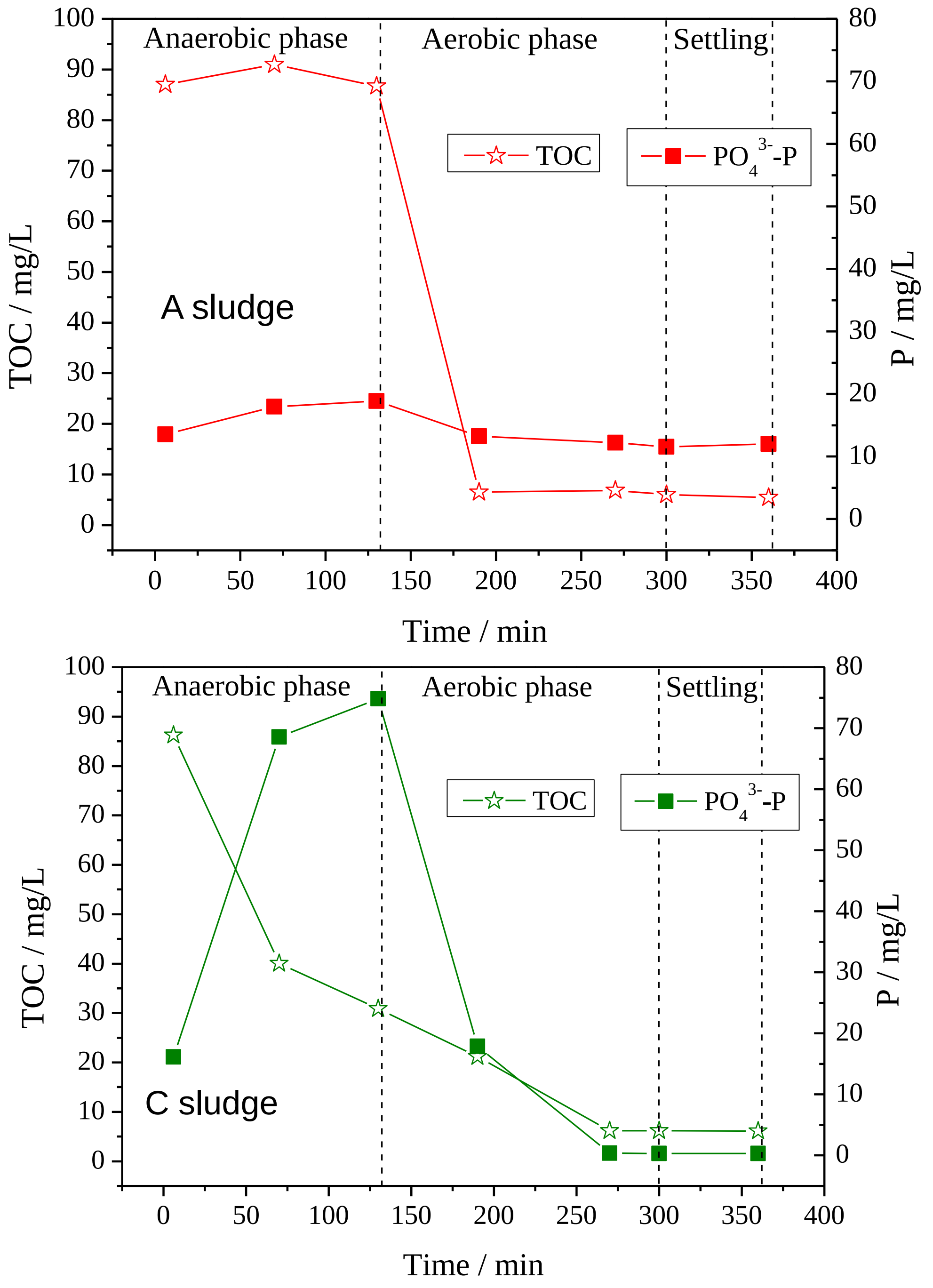

S1 Fig. Phosphorus removal and TOC removal of the sequencing batch reactor.

Sludge samples from A and C were collected for metagenomic sequencing.
