# Supplementary material for: Exploring the Shift in Structure and Function of Microbial Communities Performing Biological Phosphorus Removal
Source: PLoS One. 2016 Aug 22;11(8):e0161506. doi: 10.1371/journal.pone.0161506 (PMC4993488; doi:10.1371/journal.pone.0161506)
Supplement: S2 Fig — (PDF) [file pone.0161506.s002.pdf]

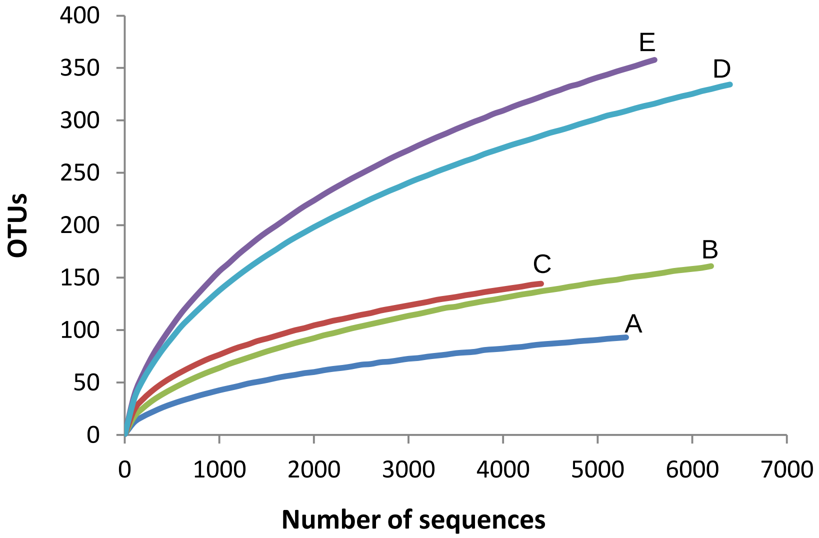

S2 Fig. Rarefaction curves of sludge samples for 16S rRNA gene pyrosequencing with 97% similarity. The rarefaction curve, plotting the number of observed OTUs as a function of the number of clean sequences (pyro-tags), was clustered by using OTU-based methods integrated in Mothur (v. 1.33.2).
