# Supplementary material for: Exploring the Shift in Structure and Function of Microbial Communities Performing Biological Phosphorus Removal
Source: PLoS One. 2016 Aug 22;11(8):e0161506. doi: 10.1371/journal.pone.0161506 (PMC4993488; doi:10.1371/journal.pone.0161506)
Supplement: S3 Fig — (PDF) [file pone.0161506.s003.pdf]

	Group	A	B	C	D	E	Class
	OTU0001	19%	64%	35%	0%	0%	*Alphaproteobacteria*
	OTU0002	47%	6%	4%	4%	17%	*Betaproteobacteria*
	OTU0003	0%	0%	4%	16%	5%	*Bacilli*
	OTU0004	0%	0%	12%	0%	0%	*Alphaproteobacteria*
	OTU0005	0%	0%	0%	1%	23%	*Gammaproteobacteria*
**Accumulibacter** →	OTU0006	0%	3%	9%	0%	8%	*Betaproteobacteria*
	OTU0007	0%	0%	0%	10%	5%	*Alphaproteobacteria*
	OTU0008	16%	0%	0%	0%	0%	*Betaproteobacteria*
	OTU0009	0%	0%	1%	9%	1%	*Flavobacteria*
	OTU0010	0%	0%	0%	5%	4%	*Gammaproteobacteria*
	OTU0011	0%	5%	1%	0%	0%	*Flavobacteria*
	OTU0012	0%	0%	0%	8%	0%	TM7 genera incertae sedis
	OTU0013	0%	4%	2%	0%	0%	*Acidobacteria* Gp4
	OTU0014	0%	0%	0%	6%	0%	TM7 genera incertae sedis
	OTU0015	0%	0%	2%	0%	3%	*Sphingobacteria*
	OTU0016	1%	2%	1%	0%	0%	*Betaproteobacteria*
	OTU0017	0%	0%	0%	3%	2%	*Flavobacteria*
	OTU0018	1%	2%	0%	0%	0%	*Betaproteobacteria*
	OTU0019	0%	3%	1%	0%	0%	*Gammaproteobacteria*
	OTU0020	0%	0%	1%	2%	1%	*Actinobacteria*
	OTU0021	0%	0%	0%	1%	2%	*Gammaproteobacteria*
	OTU0022	2%	1%	0%	0%	0%	*Acidobacteria* Gp4
	OTU0023	0%	1%	1%	0%	0%	*Sphingobacteria*
	OTU0024	2%	0%	0%	0%	0%	*Alphaproteobacteria*
	OTU0025	0%	0%	1%	0%	0%	*Flavobacteria*
	OTU0026	0%	0%	1%	0%	0%	*Flavobacteria*
**Accumulibacter** →	OTU0027	0%	0%	0%	1%	2%	*Betaproteobacteria*
	OTU0028	0%	0%	1%	0%	0%	*Acidobacteria* Gp4
	OTU0029	0%	0%	1%	0%	0%	*Betaproteobacteria*
	OTU0030	0%	0%	0%	2%	0%	unclassified *Bacteroidetes*
	OTU0031	0%	0%	0%	0%	2%	*Alphaproteobacteria*
	OTU0032	0%	0%	2%	0%	0%	unclassified_*Bacteroidetes*
	OTU0033	0%	1%	1%	0%	0%	*Acidobacteria*_Gp4
	OTU0034	0%	0%	1%	0%	0%	unclassified_*Bacteroidetes*
	OTU0035	0%	0%	0%	2%	0%	TM7_genera_incertae_sedis
	OTU0036	0%	0%	0%	2%	0%	*Gammaproteobacteria*
	OTU0037	0%	0%	1%	0%	0%	*Sphingobacteria*
	OTU0038	0%	0%	1%	0%	0%	*Gammaproteobacteria*
	OTU0039	1%	0%	0%	0%	0%	*Gemmatimonadetes*
	OTU0040	0%	0%	1%	0%	0%	*Alphaproteobacteria*
	Sum	91%	92%	87%	71%	73%	

S3 Fig. Relative abundances of bacterial populations in the dominant classes at different stages of the SBR. Taxonomic classification was conducted by assigning 16S rRNA pyro-tags against SILVA SSU Ref database (v. 115) by BLASTN (v. 2.2.29+) with *e*-value cutoff of 1e-20.
