# Supplementary material for: Exploring the Shift in Structure and Function of Microbial Communities Performing Biological Phosphorus Removal
Source: PLoS One. 2016 Aug 22;11(8):e0161506. doi: 10.1371/journal.pone.0161506 (PMC4993488; doi:10.1371/journal.pone.0161506)
Supplement: S4 Fig — Rhodocyclus tenuis was recruited as the out-group species. (PDF) [file pone.0161506.s004.pdf]

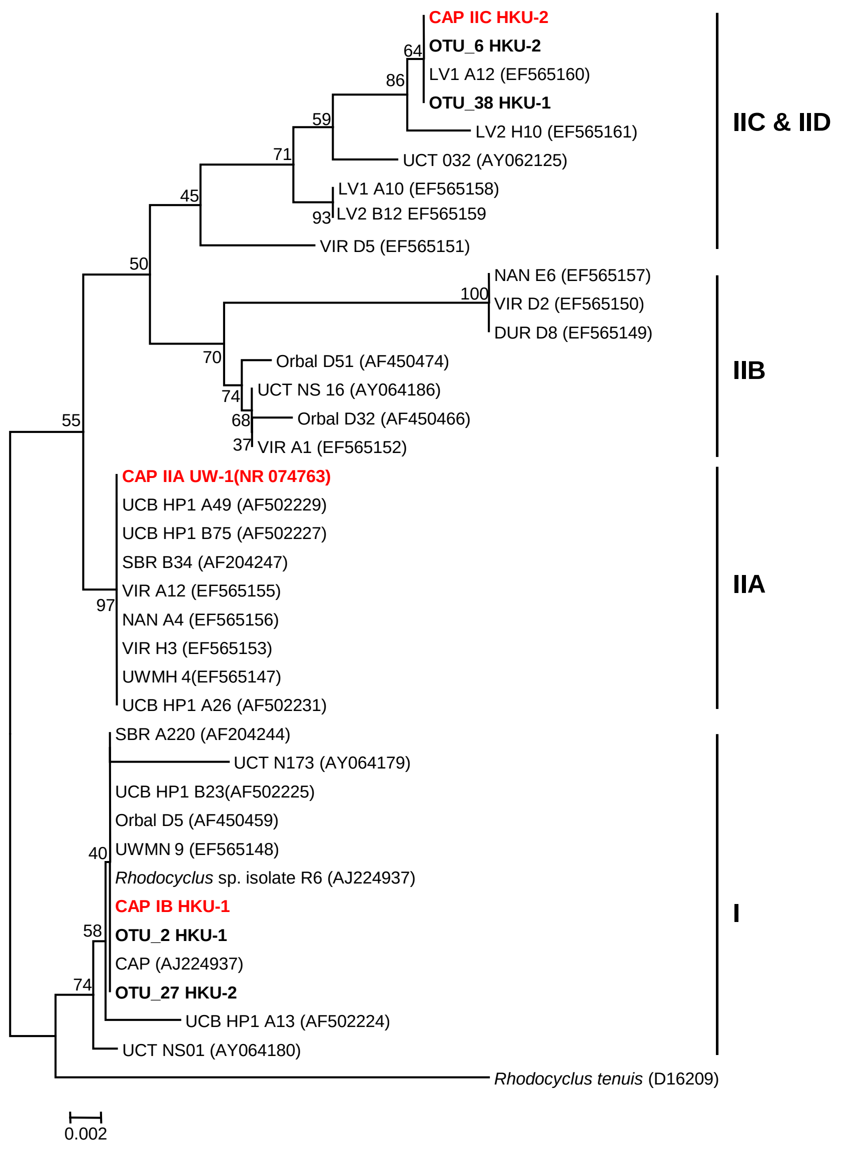

S4 Fig. Phylogenetic tree of 16S rRNA genes from Accumulibacter draft genomes, representative pyro-tags of the OTU groups assigned to Accumulibacters and reference sequences downloaded from GenBank. *Rhodocyclus tenuis* was recruited as the out-group species. The 16S rRNA genes of OTU_38 HKU-1, OTU_2 HKU-1 and CAP IB HKU-1 were downloaded from the published paper [13].

References in Supporting Information

13. Mao Y, Yu K, Xia Y, Chao Y, Zhang T. Genome reconstruction and gene expression of "*Candidatus* Accumulibacter phosphatis" Clade IB performing biological phosphorus removal. Environ Sci Technol. 2014;48(17):10363-71. doi: 10.1021/es502642b.
