# Supplementary material for: Exploring the Shift in Structure and Function of Microbial Communities Performing Biological Phosphorus Removal
Source: PLoS One. 2016 Aug 22;11(8):e0161506. doi: 10.1371/journal.pone.0161506 (PMC4993488; doi:10.1371/journal.pone.0161506)
Supplement: S5 Fig — (PDF) [file pone.0161506.s005.pdf]

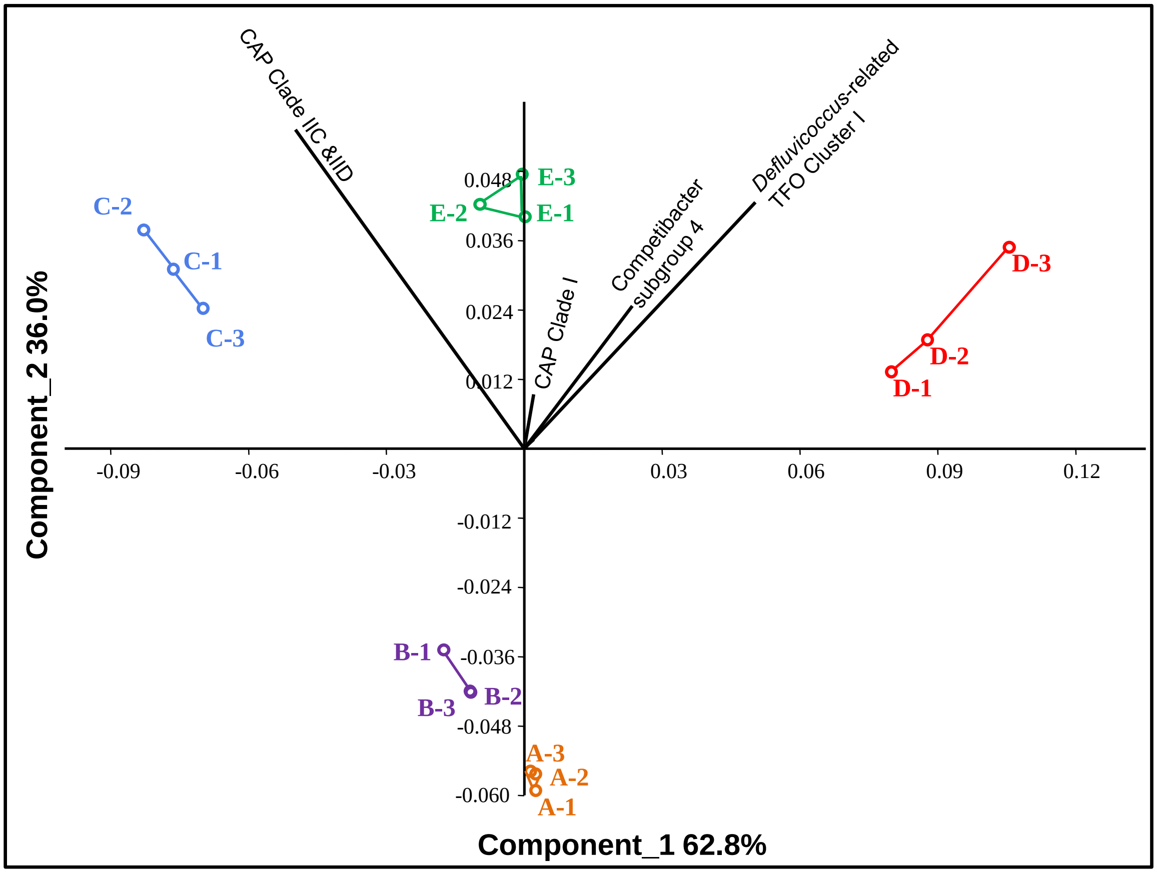

S5 Fig. Principle components analysis of five sludge samples extracted from the SBR according to bacterial abundance involved in EBPR process. Abundance of specific PAO and GAO subgroup is estimated by the number of pyro-tags assigned to representative 16S rRNA genes. Each sludge sample has triplicate pyro-tag datasets which are indicated by circles and linked by the line.
