# Supplementary material for: Exploring the Shift in Structure and Function of Microbial Communities Performing Biological Phosphorus Removal
Source: PLoS One. 2016 Aug 22;11(8):e0161506. doi: 10.1371/journal.pone.0161506 (PMC4993488; doi:10.1371/journal.pone.0161506)
Supplement: S6 Fig — (PDF) [file pone.0161506.s006.pdf]

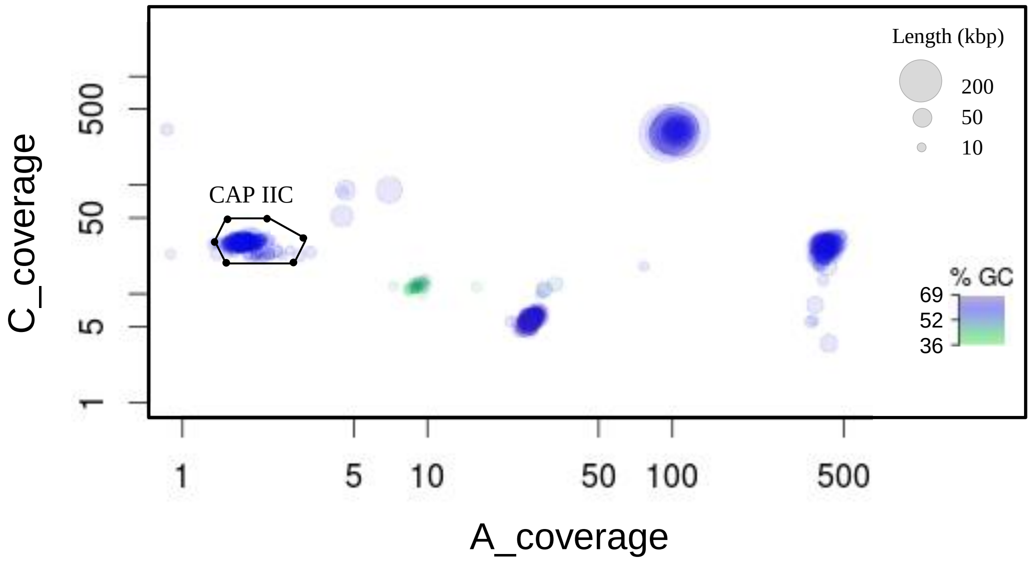

S6 Fig. Binning of CAP IIC HKU-2 draft genome by using differential coverage of metagenomics data sets from sludges A and C. Each circle represents a scaffold, with size proportional to length and colored by guanine-cytosine (GC) content. Only scaffolds ≥ 10 kbp are shown.
