# Supplementary material for: Exploring the Shift in Structure and Function of Microbial Communities Performing Biological Phosphorus Removal
Source: PLoS One. 2016 Aug 22;11(8):e0161506. doi: 10.1371/journal.pone.0161506 (PMC4993488; doi:10.1371/journal.pone.0161506)
Supplement: S7 Fig — (PDF) [file pone.0161506.s007.pdf]

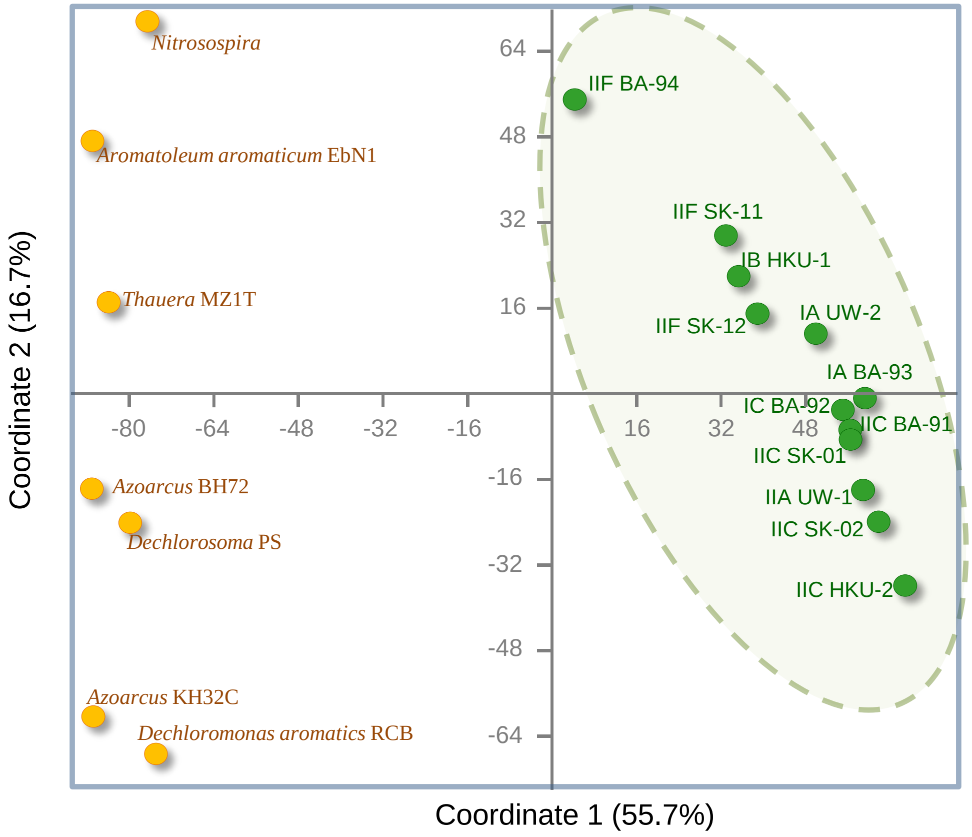

S7 Fig. Principal coordinates analysis of Accumulibacter genomes (green dots) and its six neighboring finished genomes (orange dots) in family of *Rhodocyclaceae* using the Euclidean similarity metric based upon their annotated COG categories.
