# Supplementary material for: Exploring the Shift in Structure and Function of Microbial Communities Performing Biological Phosphorus Removal
Source: PLoS One. 2016 Aug 22;11(8):e0161506. doi: 10.1371/journal.pone.0161506 (PMC4993488; doi:10.1371/journal.pone.0161506)
Supplement: S8 Fig — (PDF) [file pone.0161506.s008.pdf]

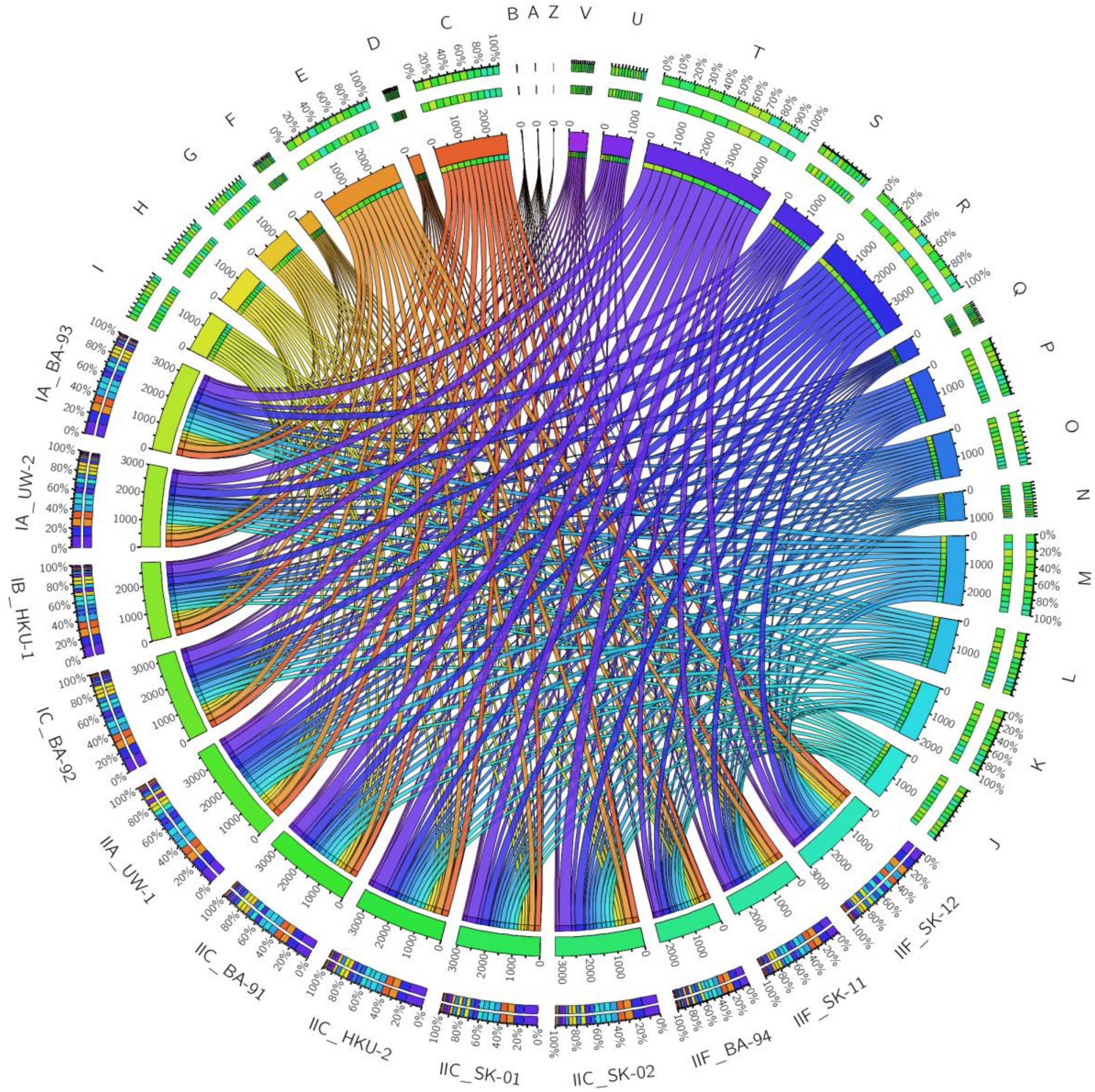


**S8 Fig. COG profile of the 12 Accumulibacter genomes.** Each gene inventory of the Accumulibacter genomes were mapped to functional categories in COG database respectively. Abbreviations of the functional categories: A indicates RNA processing and modification; B indicates chromatin structure and dynamics; C indicates energy production and conversion; D indicates cell cycle control, cell division, chromosome partitioning; E indicates amino acid transport and metabolism; F indicates nucleotide transport and metabolism; G indicates

carbohydrate transport and metabolism; H indicates coenzyme transport and metabolism; I indicates lipid transport and metabolism; J indicates translation, ribosomal structure and biogenesis; K indicates transcription; L indicates replication, recombination and repair; M indicates cell wall/membrane/envelope biogenesis; N indicates cell motility; O indicates posttranslational modification, protein turnover, chaperones; P indicates inorganic ion transport and metabolism; Q indicates secondary metabolites biosynthesis, transport and catabolism; R indicates general function prediction only; S indicates function unknown; T indicates signal transduction mechanisms; U indicates intracellular trafficking, secretion, and vesicular transport; V indicates defense mechanisms; Z indicates cytoskeleton.
