# Supplementary material for: Exploring the Shift in Structure and Function of Microbial Communities Performing Biological Phosphorus Removal
Source: PLoS One. 2016 Aug 22;11(8):e0161506. doi: 10.1371/journal.pone.0161506 (PMC4993488; doi:10.1371/journal.pone.0161506)
Supplement: S1 Table — (PDF) [file pone.0161506.s009.pdf]

S1 Table Reference 16S rRNA gene sequences of representative PAOs.

PAO	Clade	Accession NO.	Ref.	PAO	Clade	Accession NO.	Ref.
Accumulibacter	I	AF204244	[1]	*Tetrasphaera*	I	AB030911	[2-4]
		AF255641				AB072496	
		AF502224				AF387307	
		AF502225				AF387308	
		AJ224937				AF387311	
		AY064178				GU552251	
		AY064179				GU552252	
		EF565148				GU552253	
	IIA	AF204247				GU552254	
		AF502227				NR024735	
		AF502229			II	AF125091	
		AF502230				DQ007321	
		AF502231				GU552259	
		EF565147				Y14595	
		EF565153				X85211	
		EF565155				X85212	
		EF565156				Y14597	
	IIB	EF565149				AF125090	
		EF565150				NR024975	
		EF565152			III	GU552249	
		EF565157				GU552250	
	IIC & IID	AY062125				GU552255	
		EF565151				GU552257	
		EF565158				GU552260	
		EF565159				GU552263	
		EF565160				GU552265	
		EF565161				GU552268	
Candidatus Halomonas phosphatis		JN242764-JN242812	[5]	*Tetrasphaera japonica*		NR024976	
Gemmatimonas aurantiaca		AB072735	[6]			AF125092	

References in Supporting Information

1. He S, Gall DL, McMahon KD. "*Candidatus* Accumulibacter" population structure in enhanced biological phosphorus removal sludges as revealed by polyphosphate kinase genes. Appl Environ Microbiol. 2007;73(18):5865-74. doi: 10.1128/aem.01207-07.
2. Hanada S, Liu W-T, Shintani T, Kamagata Y, Nakamura K. *Tetrasphaera elongata* sp. nov., a polyphosphate-accumulating bacterium isolated from activated sludge. Int J Syst Evol Microbiol. 2002;52(3):883-7. doi: 10.1099/ijs.0.01990-0.
3. Kristiansen R, Nguyen HT, Saunders AM, Nielsen JL, Wimmer R, Le VQ, et al. A metabolic model for members of the genus *Tetrasphaera* involved in enhanced biological phosphorus removal. ISME J. 2013;7(3):543-554. doi: 10.1038/ismej.2012.136.
4. Nguyen HTT, Le VQ, Hansen AA, Nielsen JL, Nielsen PH. High diversity and abundance of putative polyphosphate-accumulating *Tetrasphaera*-related bacteria in activated sludge systems. FEMS Microbiol Ecol. 2011;76(2):256-67. doi: 10.1111/j.1574-6941.2011.01049.x.
5. Nguyen HT, Nielsen JL, Nielsen PH. '*Candidatus* Halomonas phosphatis', a novel polyphosphate-accumulating organism in full-scale enhanced biological phosphorus removal plants. Environ Microbiol. 2012;14(10):2826-37. doi: 10.1111/j.1462-2920.2012.02826.x.
6. Zhang H. *Gemmatimonas aurantiaca* gen. nov., sp. nov., a Gram-negative, aerobic, polyphosphate-accumulating microorganism, the first cultured representative of the new bacterial phylum *Gemmatimonadetes* phyl. nov. Int J Syst Evol Microbiol. 2003;53(4):1155-63. doi: 10.1099/ijs.0.02520-0.
