# Supplementary material for: Exploring the Shift in Structure and Function of Microbial Communities Performing Biological Phosphorus Removal
Source: PLoS One. 2016 Aug 22;11(8):e0161506. doi: 10.1371/journal.pone.0161506 (PMC4993488; doi:10.1371/journal.pone.0161506)
Supplement: S2 Table — (PDF) [file pone.0161506.s010.pdf]

S2 Table Reference 16S rRNA gene sequences of representative GAOs.

GAO	Subgroup	Accession NO.	Ref.	GAO	Cluster	Accession NO.	Ref.
Competibacter	1	AF361095	[7, 8]	*Defluvicoccus*-related TFO	I	AF179678	[9-12]
		AF361096				AY351635	
	2	AY098896				AY351636	
	3	AF361089				AY351638	
		AF361090				AY351639	
		AF361091				AY351640	
		AF361092				AY351641	
		AF361093				AY351643	
		AF361094				FJ356059	
	4	AF314424			II	AF527585	
		AY098897				AF527587	
		AY098898				DQ146465	
		AY098902				DQ146468	
	5	AY098900				DQ413102	
		AY098909				EU332800	
		AY098910				EU332810	
		AY098911				EU834764	
	6	AY098899			III	AB445107	
		AY098905				AB445108	
		AY098906				AB445109	
		AY098912				AB445110	
	7	AY098901				AF280850	
		AY098903			IV	AM420239	
		AY098904				DQ129305	
		AY098907				DQ413109	
		AY098908				EU834757	
		AY098913				FJ516933	

References in Supporting Information

7. Crocetti GR, Banfield JF, Keller J, Bond PL, Blackall LL. Glycogen-accumulating organisms in laboratory-scale and full-scale wastewater treatment processes. Microbiology. 2002;148:3353-64.
8. Kong Y, Ong SL, Ng WJ, Liu W-T. Diversity and distribution of a deeply branched novel proteobacterial group found in anaerobic-aerobic activated sludge processes. Environ Microbiol. 2002;4(11):753-7.
9. Meyer RL. Putative glycogen-accumulating organisms belonging to the *Alphaproteobacteria* identified through rRNA-based stable isotope probing. Microbiology. 2006;152(2):419-29. doi: 10.1099/mic.0.28445-0.
10. Wong MT, Tan FM, Ng WJ, Liu WT. Identification and occurrence of tetrad-forming *Alphaproteobacteria* in anaerobic-aerobic activated sludge processes. Microbiology. 2004;150(Pt 11):3741-8. doi: 10.1099/mic.0.27291-0.
11. Mcllroy S, Seviour RJ. Elucidating further phylogenetic diversity among the *Defluviicoccus*-related glycogen-accumulating organisms in activated sludge. Environ Microbiol Rep. 2009;1(6):563-8. doi: 10.1111/j.1758-2229.2009.00082.x.
12. Nielsen AT, Liu W-T, Filips C, Grady L, Molin JS, Stahl DA. Identification of a novel group of bacteria in sludge from a deteriorated biological phosphorus removal reactor. Appl Environ Microbiol. 1999;65:1251-8.
