# Supplementary material for: Exploring the Shift in Structure and Function of Microbial Communities Performing Biological Phosphorus Removal
Source: PLoS One. 2016 Aug 22;11(8):e0161506. doi: 10.1371/journal.pone.0161506 (PMC4993488; doi:10.1371/journal.pone.0161506)
Supplement: S3 Table — (PDF) [file pone.0161506.s011.pdf]

S3 Table Effective 16S rRNA gene pyrosequencing reads generation and microbial diversity metrics.

Sample	A-1	A-2	A-3	B-1	B-2	B-3	C-1	C-2	C-3	D-1	D-2	D-3	E-1	E-2	E-3
Number of raw reads	26,745	5,626	9,965	8,405	9,613	6,335	24,061	5,069	4,469	5,877	6,100	7,355	6,921	6,629	6,566
Number of pyro-tags [a]	5,305	5,579	9,804	8,319	9,406	6,276	13,461	5,015	4,402	5,646	5,815	6,844	6,700	6,453	6,495
Ace	71	246	224	392	287	316	181	362	312	499	606	836	491	597	689
Chao1	64	194	207	308	262	256	183	294	260	489	538	674	476	507	554
Good's coverage	100%	99%	99%	99%	99%	99%	100%	99%	98%	98%	98%	97%	98%	98%	98%

[a] The pyro-tags were generated after the raw reads were denoised by AmpliconNoise (implemented in QIIME v.1.3.0) and Chimera filtering using Chimera Slayer
