# Supplementary material for: Exploring the Shift in Structure and Function of Microbial Communities Performing Biological Phosphorus Removal
Source: PLoS One. 2016 Aug 22;11(8):e0161506. doi: 10.1371/journal.pone.0161506 (PMC4993488; doi:10.1371/journal.pone.0161506)
Supplement: S1 Text — (PDF) [file pone.0161506.s012.pdf]

S1 Text Supplementary Materials and Methods

Chemical analysis

Orthophosphate-phosphorus (PO_4^{3-}–P) was measured by ascorbic acid method after the solution being filtered by the membrane with 0.45 μm pore size. Total phosphorus (TP) was measured by ascorbic acid method after digestion with potassium persulfate. The total organic carbon (TOC) in each sample was determined by using a TOC analyzer (Shimadzu TOC-5000, Japan). All the samples for TOC measurement were filtered through the membrane with 0.45 μm pore size before analyzing. The volatile suspended solids (VSS) were tested according to the standard methods [14].

Diversity of Accumulibacter

A designed primer set (ACCppk1-254F: TCACCACCGACGGCAAGAC and ACCppk1-1376R: ACGATCATCAGCATCTTGGC) targeting the Accumulibacter cluster [15] was used to amplify the *ppk*1 gene fragments from the two DNA samples from sludges A and C. Triplicate 50 μl PCR amplification solutions were prepared for each sample, containing 25 μl of Premix Ex Taq™ (TaKaRa, Dalian, China), 1 μl of 10 μM forward and reverse primers, 50 ng of extracted DNA, and water (RT-PCR grade, Ambion Inc., USA). The thermocycling steps for PCR were set as follows: initial denaturation at 95 °C for 4 min followed by 30 cycles of denaturation at 95 °C for 30 s, annealing at 65 °C for 1min, and extension at 72 °C for 2 min; and a final extension step at 72 °C for 12 min. The amplified fragments were used for the clone library and then sequencing of positive clones.

References in Supporting Information

14. APHA. Standard methods for the examination of water and wastewater 20th edition. American Public Health Association, Washington DC. 2005.
15. McMahon KD, Yilmaz S, He S, Gall DL, Jenkins D, Keasling JD. Polyphosphate kinase genes from full-scale activated sludge plants. Appl Microbiol Biotechnol. 2007;77(1):167-73. doi: 10.1007/s00253-007-1122-6.
